# Discovery of Novel Non-Steroidal Cytochrome P450 17A1 Inhibitors as Potential Prostate Cancer Agents

**DOI:** 10.3390/ijms21144868

**Published:** 2020-07-09

**Authors:** Tomasz M. Wróbel, Oksana Rogova, Kasper L. Andersen, Rahul Yadav, Simone Brixius-Anderko, Emily E. Scott, Lars Olsen, Flemming Steen Jørgensen, Fredrik Björkling

**Affiliations:** 1 Department of Drug Design and Pharmacology, University of Copenhagen, Universitetsparken 2, DK-2100 Copenhagen, Denmark; oksana.rogova@chem.lu.se (O.R.); lo@sund.ku.dk (L.O.); fsj@sund.ku.dk (F.S.J.); fb@sund.ku.dk (F.B.); 2 Department of Synthesis and Chemical Technology of Pharmaceutical Substances, Medical University of Lublin, Chodźki 4a, 20093 Lublin, Poland; 3 Biotech Research and Innovation Centre (BRIC), University of Copenhagen, Ole Maaløes Vej 5, DK-2200 Copenhagen, Denmark; kasper.andersen@bric.ku.dk; 4 Department of Medicinal Chemistry, University of Michigan, 428 Church Street, Ann Arbor, MI 48109-1065, USA; ry205@msstate.edu (R.Y.); sbrixius@med.umich.edu (S.B.-A.); scottee@med.umich.edu (E.E.S.); 5 Department of Pharmacology, University of Michigan, 428 Church Street, Ann Arbor, MI 48109-1065, USA; 6 Protein Engineering, Novozymes A/S, Krogshøjvej 36, DK-2880 Bagsvaerd, Denmark

**Keywords:** cytochrome P450 17A1, CYP17A1, prostate cancer, enzyme inhibition

## Abstract

The current study presents the design, synthesis, and evaluation of novel cytochrome P450 17A1 (CYP17A1) ligands. CYP17A1 is a key enzyme in the steroidogenic pathway that produces androgens among other steroids, and it is implicated in prostate cancer. The obtained compounds are potent enzyme inhibitors (sub µM) with antiproliferative activity in prostate cancer cell lines. The binding mode of these compounds is also discussed.

## 1. Introduction

**Figure 1:**
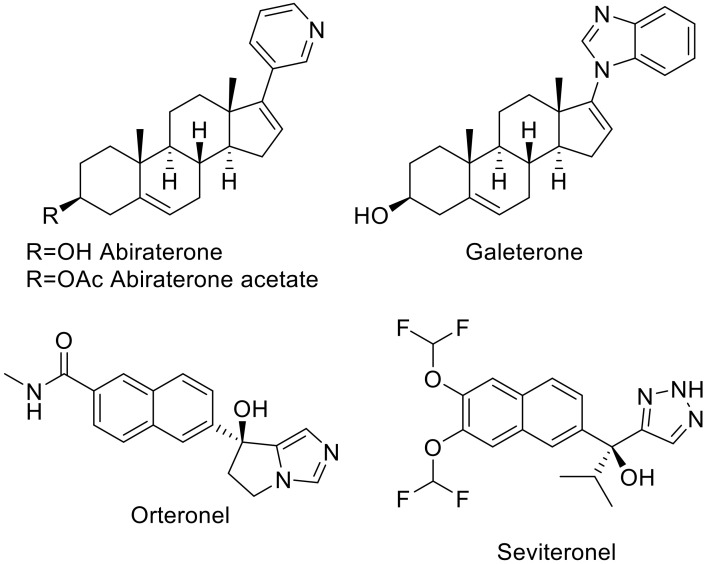
Structures of the cytochrome P450 17A1 inhibitors.

Prostate cancer (PCa) is expected to be the most frequently diagnosed cancer in 2020 and also the second deadliest in men [1]. The link between the disease and the production of male hormones has been established since 1941 and treatments aimed at blocking the synthesis of these hormones, so called androgen deprivation therapies (ADT), were subsequently developed for clinical use [2]. A notable example involves usage of gonadotropin releasing hormone (GnRH) agonists and antagonists to reduce serum testosterone levels known as “chemical castration” [3]. Unfortunately, resistance to ADT occurs within a short period of time and PCa evolves into castration resistant prostate cancer (CRPC), in which the cancer cells produce androgens via de novo and “back door” pathways [4,5]. Previous treatments developed against CRPC, such as chemotherapy with docetaxel in combination with prednisone, only have limited therapeutic efficacy and their use was associated with severe side effects including anaemia and hepatotoxicity [6]. Thus, suppression of hormone levels remains the key objective in the treatment of advanced prostate cancer. The cytochrome P450 17A1 (CYP17A1) enzyme is required for androgen production via both the de novo and “back-door” pathways and has been identified as the most promising target to block androgen synthesis [2]. Accordingly, both academic and industrial research groups have focused on the development of CYP17A1 inhibitors, with such efforts leading to the approval of abiraterone acetate (Figure 1) for the treatment of CRPC by the Food and Drug Administration (FDA) and the European Medicines Agency (EMA) in 2011.

Abiraterone (ABT), an FDA approved drug, was designed based on the pregnenolone structure with a pyridyl substituent in position 17 [7]. Other discovery efforts include development of the abiraterone analogue galeterone (discontinued after phase III clinical trials), and the nonsteroidal compounds S-orteronel/TAK-700 [8] (terminated after phase III trials) and S-seviteronel/VT 464 [9] (completed phase II trials).

Crystal structures of CYP17A1 complexed with the steroidal inhibitors reveal that in each case an sp^2^ hybridized nitrogen atom on a substituent extending from C17 coordinates directly to the heme iron [10,11]. In abiraterone and galeterone, the coordinating substituents are pyridine and benzimidazole respectively. Their steroid cores form a 60° angle with the heme plane and both substrates and inhibitors have the distal, C3 end of the steroid interacting with Asn202 [10].

**Figure 2:**
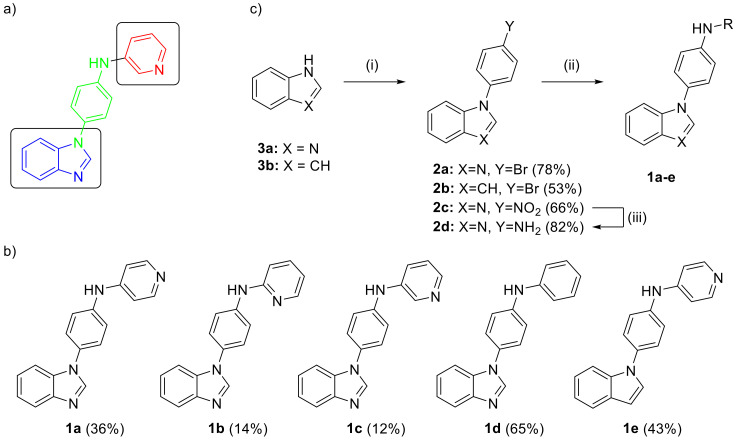
Compounds in the present study (**a**) highlighting the pyridine and benzimidazole moieties connected with the aniline linker (**b**) full structures and isolated yields (**c**) synthesis scheme with reagents and conditions: (i) 1-bromo-4-fluorobenzene or 1-fluoro-4-nitrobenzene, K_3_PO_4_, DMF, 150 °C, (ii) ^t^BuXPhos Pd G1/G3, ^t^BuXPhos, amine or 3-bromopyridine, ^t^BuONa, ^t^BuOH, RT to 70 °C, (iii) 10% Pd/C, MeOH, RT.

In our efforts towards the discovery of novel non-steroidal ligands, we wanted to investigate compounds that have the possibility of binding either with the pyridine or benzimidazole moiety interacting with the heme group by combining both moieties into the same structure (Figure 2a). Linking those two moieties with benzene ring provided the most versatile way to reveal structures which would be similar in size and shape to abiraterone, so that they could be expected to occupy the same binding pocket. These compounds were assessed for their binding affinity and inhibition of purified CYP17A1 enzyme and their antiproliferative activity in selected prostate cancer cell lines.

## 2. Results and Discussion

### 2.1. Synthesis

Simple non-steroidal analogues were designed which would allow exploration of the effect of a different substitution pattern of pyridine on enzymatic activity. Compounds where the benzimidazole fragment was replaced with indole (**1e**) and the pyridyl group replaced with phenyl (**1d**) were also synthesized to allow only one part of a molecule to be able to interact with the heme group. Thus, five novel compounds (Figure 2b) were prepared where the aniline linker was selected to facilitate synthesis and provide a symmetrical linker with two nitrogen atoms para to each other. The final compounds were obtained via the Buchwald–Hartwig cross-coupling of heterocyclic bromides with corresponding amines, providing a most versatile route to the target compounds [12] (Supplementary Materials S1). Starting benzimidazole (**3a**) or indole (**3b**) was arylated by nucleophilic aromatic substitution with 4-bromofluorobenzene to provide *N*-substituted bromides (**2a** and **2b**) (Figure 2c) [13]. The subsequent C-N bond formation in compounds **1a**, **1b**, **1d,** and **1e** was then performed with the target aminopyridine or aniline amino groups. After evaluating several pre-catalysts, ^t^BuXPhos Pd was selected for this transformation. Compound **1c** was approached differently taking advantage of the inhouse availability of 3-bromopyridine. This coupling required reversing polarity during Buchwald-Hartwig reaction which was enabled by employing aniline **2d**. This aniline derivative was obtained from catalytic hydrogenation of the corresponding nitro compound (**2c**), which was prepared similarly to the bromo analogues.

### 2.2. Enzyme Inhibition

The target compounds were initially tested for binding mode and affinity for CYP17A1 using a spectral binding assay. This assay can distinguish the mode of interaction, with a blue shift of the Soret absorbance occurring when ligand displaces water from the heme iron to generate a five-coordinate iron without direct coordination to the heme iron (type I, similar to most substrates) or a red shift of the Soret peak when the ligand nitrogen replaces the water to directly interact with the six-coordinate iron (type II, similar to many inhibitors) [14]. All compounds herein displayed type II binding, consistent with direct binding between the ligand nitrogen and heme iron (Supplementary Materials S2). Comparison of compounds **1a**–**1c** indicated that the position of the pyridine nitrogen atom is important, with **1c** containing 3-pyridyl (Kd 96 nM) having a 2–3-fold higher affinity than the 2-pyridyl (Kd 420 nM) or 4-pyridyl (Kd 290 nM) analogues (Table 1). This observation suggests that 3-pyridyl fragment is important for optimal ligand alignment. This 3-pyridyl fragment is also found in abiraterone. Interestingly, only somewhat reduced affinity for CYP17A1 was observed for compound **1d**, in which the pyridine is replaced by a phenyl ring and compound **1e**, in which the benzimidazole moiety was replaced with indole (Table 1). This indicates that either end of the molecule appears to be able to coordinate the iron atom.

Regardless of their binding affinity, all compounds were tested for their ability to inhibit progesterone 17α-hydroxylation by recombinant purified CYP17A1 (Supplementary Materials S2). Consistent with the low affinity of compounds **1a** and **1b,** they had high IC_50_ values, 8 µM and >10 µM, respectively. The higher affinity compounds **1c**, **1d**, and **1e** were more potent with IC_50_ values of 0.83 µM, 1.76 µM, and 0.56 µM, respectively.

Since iron-binding heterocycles have the potential to bind and inhibit other human cytochrome P450 enzymes with similar active site topology, we tested the two most potent CYP17A1 inhibitors, **1c** and **1e**, against the important drug-metabolizing enzymes CYP3A4 and CYP2D6. In human drug metabolism, CYP3A4 and CYP2D6 are dominant enzymes and undesired inhibition could potentially alter the pharmacokinetics and reduce the clearance of co-administered drugs. CYP3A4 is particularly important in this respect, with a large and flexible active site able to accommodate many different molecular scaffolds including steroids. At concentrations up to 10 µM, neither compound inhibited CYP3A4 nifedipine metabolism by 50% (IC_50_ > 10 µM). Similarly, compound **1c** poorly inhibited CYP2D6 dextromethorphan metabolism (IC_50_ > 10 µM). Compound **1e** did demonstrate moderate inhibition of CYP2D6 (IC_50_ = 2.5 µM), but this was 4.5-fold weaker than inhibition of CYP17A1. The most similar steroidogenic human enzyme to CYP17A1 is CYP21A2, which also uses progesterone as a substrate, in this case to form 21-hydroxyprogesterone. Undesired inhibition of this enzyme could adversely impact the biosynthesis of the corticosteroids cortisol and aldosterone which control stress/immune responses and blood pressure, respectively [15]. Abiraterone itself has this liability [16] and is thus co-administered with the synthetic corticosteroid prednisone [15]. Compound **1e** potently inhibited CYP21A2 with a sub micromolar IC_50_ (IC_50_ = 0.19 µM). Combined with moderate inhibition of CYP2D6, these results suggest that compound **1e** has the potential to yield significant off-target effects. While compound **1c** did not demonstrate significant inhibition of CYP3A4 or CYP2D6, it inhibited CYP21A2 with an IC_50_ (1.5 µM) only two-folder higher than CYP17A1 (0.83 µM). This finding suggests that retaining the benzimidazole moiety would be at least partly beneficial for the compound selectivity. However, at the same time it might be possible to achieve similar results by altering the nitrogen atom position in the pyridine fragment in compound **1e**. Further optimization of **1c** is required to eliminate off-target effects on the steroidogenic human CYP21A2 enzyme to prevent disruption of glucocorticoid and mineralocorticoid biosynthesis in vivo. This might be achieved by adding substituents compatible with the CYP17A1 active site topography forming interactions with the bordering Arg239 and/or Asp298 residues, but incompatible with the more spatially constrained CYP21A2 active site, as has been accomplished for abiraterone [17].

### 2.3. Antiproliferative Activity

**Figure 3:**
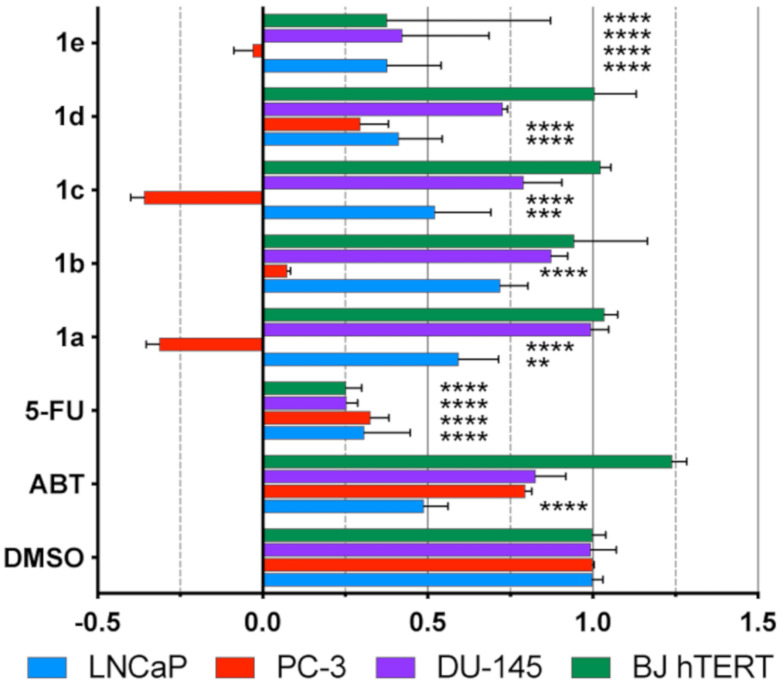
Growth rate inhibition (GR) of 5-fluorouracil (5-FU), Abiraterone (ABT) and compounds **1a**-**e**. All compounds were tested at 10 µM, with DMSO as a control; The sign of the GR value relates directly to response phenotype: Values between 0 and 1 show partial growth inhibition, a value of 0 equals cytostasis, and values between 0 and −1 show compounds are cytotoxic. Dunnett’s test used for multiple comparison to a single control. The stars signify an adjusted *p* value: ** *p* < 0.01; *** *p* < 0.001; **** *p* < 0.0001.

Next, we tested the effect of compounds **1a**–**1e** on prostate cancer derived cell lines. The anti-proliferative activity was determined in vitro by adding 10 µM compound to each of three prostate cancer cell lines (LNCaP, DU145, and PC3). As an additional comparison, immortalized non-cancerous fibroblasts (BJ hTERT) were used. These cancer cell lines are considered the classic standard of PCa cell culture [18]. Since these cell lines grew at very different rates, inhibition of growth rate (GR) was used as a metric [19]. All compounds except **1e** were non-toxic to non-cancerous cells. Compounds **1a** and **1c** were both cytotoxic in the PC3 cell line (Figure 3). The other compounds caused >50% growth inhibition in this cell line. DU145 cells were weakly affected by only compound **1e** with growth rate inhibition more than 50% (GR < 0.5). A similar level of growth inhibition was observed for compound **1d**, **1e** in LNCaP cells, and **1b** and **1d** in PC3 cells. The reference compound abiraterone, a potent CYP17A1 inhibitor, showed low antiproliferative activity in androgen independent PC3 cells at 10 µM. These findings suggest the involvement of additional mechanisms of action. We observed substantial susceptibility of PC3 cells in comparison to DU145 (see Supplementary Materials S3 for GR_50_ in PC3 cells). These two cell lines are similar in many respects. They are both androgen independent, express lower levels of androgen receptor (AR) protein compared to LNCaP [20], do not express prostate specific antigen (PSA) or human glandular kallikrein 1 (hK1) and they have similar doubling times [21]. However, they differ in their origins, PC3 being derived from a lumbar vertebral metastatic prostate tumour and DU145 from a brain metastatic prostate tumour [21]. Notably, PC3 cells have higher metastatic potential than DU145 and LNCaP cells [22]. Autophagy is also differently induced among them with DU145 cells being particularly impaired [23]. By observing a uniform response of all cell lines used in the present study to 5-fluorouracil (5-FU), we were able to rule out any general cell dependent variability in chemosensitivity towards anti-metabolite treatment. Still, chemoresistance in PC3 and DU145 may be mediated by other mechanisms [24,25]. While AR also functions as a tumour suppressor inhibiting cell proliferation, it is possible in AR deprived cells to observe growth inhibition when cells retain necessary coregulators. Indeed, it was shown that PC3, but not DU145 retained these coregulators to achieve AR tumour suppressor function [26]. It is conceivable that our compounds can potentially influence this pathway, however no clear mechanism can be derived from this observation and further research is therefore warranted.

### 2.4. Molecular Modelling

In order to probe possible additional details in how these compounds bind to CYP17A1, all five compounds **1a**–**1e** were subjected to docking studies. For **1a** and **1c**, we observed poses with either the pyridine or benzimidazole moiety coordinating to the iron atom in the heme group. The other compounds showed predominantly only one binding pose. Compounds **1d** and **1e** with only one nitrogen atom available for coordination were positioned accordingly and compound **1b** was coordinating with benzimidazole moiety (Supplementary Materials S4).

We submitted each complex for 100 ns molecular dynamics (MD) simulations without any constraints with different initial velocities to explore if the docking poses represented the optimal binding mode. The focus of our analyses of the MD trajectories was coordination to the iron atom in the heme group and the possibility for the interactions with Arg239 and/or Asp298. Simulations of compounds **1a** and **1c**, which displayed mixed binding modes, were most revealing and generally showed a preference for pyridine-iron coordination. This is in agreement with previous DFT calculations showing stronger heme affinity towards pyridine than benzimidazole [27]. A comparison of the average free energies of binding calculated by the MM/GBSA method (Table 2) shows that compounds **1a**, **1c** and **1e** should bind better to the CYP17A1 enzymes than **1b** and **1d**. However compound **1a** suffers a penalty due to unfavourable nitrogen atom position in the pyridine ring which agrees with the experimentally determined binding (Table 1). It is also noteworthy to add that, although binding through pyridine nitrogen atom seems to be preferred, the relatively low binding energy of compound **1d** together with high affinity demonstrates that binding through benzimidazole cannot be ruled out. However, in the case of compound **1d**, there is additional interaction where NH between the benzene linker and pyridine ring forms a hydrogen bond to Asp298 and pi-cation interaction between benzene ring and the charged sidechain of Arg239.

## 3. Materials and Methods

### 3.1. Synthetic Protocols

All reagents and solvents were used as purchased from commercial sources and reactions were carried out under anhydrous and air-free conditions under inert atmosphere unless stated otherwise. Reaction conditions and yields were not optimized. Dry column vacuum chromatography (DCVC) was performed with silica gel 60 (15–40 µm, Merck KGaA, Darmstadt, Germany). Ion exchange chromatography was performed on ISOLUTE^®^ MP-TsOH columns (sulfonated macroporous polystyrene resin, 500 mg, 6 mL, Biotage, Uppsala, Sweden). Ion exchange column was first conditioned with MeOH (4 mL), then the compound dissolved in MeOH was loaded onto the column, and the column was washed with MeOH (10 mL) to remove non-basic impurities. Then, 2M solution of ammonia in MeOH (4 mL) was added to elute the compound from the column and washed with MeOH (8 mL) for complete compound recovery upon solvent removal.

### 3.2. NMR

^1^H and ^13^C spectra were recorded on 600 MHz Bruker Avance III HD or 400 MHz WB Bruker Avance spectrometers (Bruker, Billerica, MA, USA). Coupling constants (J) are reported in Hertz (Hz). Chemical shifts are reported in parts per million (ppm, δ scale) relative either to internal standard (TMS) or residual solvent peak.

### 3.3. MS

High resolution mass spectroscopy (HRMS) was carried out on a Bruker Solarix XR 7T ESI/MALDI-FT-ICR mass spectrometer (Bruker, Billerica, MA, USA) with positive MALDI ionization mode using NaTFA cluster-ions for external calibration. Data obtained were processed in Bruker DataAnalysis Software version 4.4.

### 3.4. HPLC

Analytical HPLC was carried out on Dionex UltiMate HPLC system (Thermo-Fisher, Waltham, MA, USA) consisting of LPG-3400A pump, WPS-3000SL autosampler, and DAD-3000D diode array detector using Gemini-NX C18 column (4.6 × 250 mm, 3 µm, 110 Å).

Preparative HPLC was carried out on an Dionex UltiMate HPLC system consisting of HPG-3200BX pump, Rheodyne 9725i injector, 10 mL loop, MWD-3000 detector, and FCA_Multi automated fraction collector using Gemini-NX C18 (21.2 × 250 mm, 5 µm, 110 Å).

Both analytical (1 mL/min) and preparative (21.2 mL/min) HPLC were performed with gradient elution, 0–100% solvent B (ACN-H_2_O-TFA 90:10:0.1) in solvent A (H_2_O-TFA 100:0.1) over 15 min. Data were acquired and processed using the Chromeleon Software version 6.80.

### 3.5. Spectral Binding Assay

Initial ligand binding was evaluated by measuring absorbance changes in CYP17A1 upon addition of ligands. Purified recombinant human cytochrome P450 17A1 enzyme (1 µM) in buffer (25 mM potassium phosphate, pH 7.4, 200 mM NaCl, 20% glycerol) was titrated with ligands. Ligands were dissolved in DMSO and added to three concentrations (500, 1000, and 2000 nM). The spectral intensity difference (ΔA) was measured as difference between minimal absorption (A_max_) and maximal absorption (A_min_). The A_min_ was typically at ~410 nm and A_max_ at ~440 nm for the type II changes observed for these ligands.

### 3.6. Cytochrome P450 17A1 17α-Hydroxylation Inhibition Assay

Purified recombinant human CYP17A1 (20 pmol) and human full-length cytochrome P450 reductase (80 pmol) were used to convert progesterone (purchased from Acros Organics, Fair Lawn, NJ, USA) (6 µM) to 17α-hydroxyprogesterone. Test inhibitors were dissolved in DMSO and initially tested in concentrations ranging from 1.3 to 41 µM. In subsequent assays the range of inhibitor concentrations was optimized to best define the IC_50_ of individual compounds. Reactions were run and the 17α -hydroxyprogesterone product measured by LC-MS as previously published [17].

### 3.7. Inhibition Assays for CYP3A4, CYP2D6, and CYP21A2

Selected inhibitors were tested for off-target inhibition of purified recombinant human CYP3A4 nifedipine metabolism and CYP2D6 dextromethorphan metabolism as described with minor modifications [28]. CYP3A4 nifedipine (purchased from Sigma-Aldrich, St. Louis, MO, USA) activity assays were carried out with 200 µM nifedipine for 30 min. The CYP2D6 dextrometorphan (purchased from Sigma-Aldrich, St. Louis, MO, USA) assays were carried out with 100 µM DXM. Inhibition of steroidogenic cytochrome P450 21A2 progesterone 21-hydroxylation was evaluated as reported [17].

### 3.8. Prostate Cancer Cell Line Proliferation Screening

LNCaP, PC-3, and DU-145 prostate cancer cells were propagated in RPMI-1640, GlutaMAX + 25 mM HEPES (Gibco) supplemented with 100 U/mL penicillin and 100 mg/mL streptomycin (P/S) (Gibco) and 10% (LNCaP) or 6% (PC-3 and DU-145) fetal bovine serum (FBS) (HyClone). Non-cancerous BJ fibroblast cells immortalized with expression of hTERT cells were propagated in DMEM GlutaMAX + 4.5 g/L D-glucose and pyruvate supplemented with 10% FBS and P/S.

Cells were grown to approximately 80% confluence in 150 mm culture dishes and harvested by 0.25% Trypsin/EDTA (Gibco) treatment. Released cells were counted and seeded in 384 well plates (Falcon, ref. 353962) in 30 µl media with a cell count of 1500 (BJ cells, PC-3) or and 3500 (LNCaP (Lund)) cells per well. Liquid handling steps involving cell seeding and staining were performed on a MicroLab STARlet liquid handling workstation with a CO-RE 384 probe head (Hamilton Company). The cells incubated for 24 h, after which cell counts in wells of one 384-well plate were determined as described below (time zero). In parallel, the indicated CYP compounds (10 mM stock in DMSO) or DMSO alone were delivered to wells in the remaining plates (30 nL) by acoustic droplet ejection using an Echo 550 Liquid Handler (Labcyte) for a final concentration of 10 µM compound and 0.1% DMSO. Alternatively, compound and DMSO were added in combination to achieve dilution series all with a final 0.1% DMSO concentration. Cells were then incubated for an additional 48 h. Cells were live-stained for 30 min. using 1:1200 dilutions of Höechst (Thermo Fisher Scientific 33342; 10 mg/mL) and propidium iodide (PI; 2 mg/mL) for assessment of total and dead cell numbers, respectively. Image acquisition was done using the automated microscope InCell Analyzer 2200 (GE Healthcare) with four fields (10× objective) acquired per well. Image analysis was performed using the InCell Analyzer 2200 Workstation 3.7.3 software (GE Healthcare) where nuclei were segmented by the Höechst signal (total number of cells) and the mean PI intensity in the nuclei was used to gate live and dead cells (PI-negative cells: live cells). The number of live cells per well from three independent plates were determined (compounds *n* = 3; DMSO wells *n* = 15–30) and normalized growth rate inhibition (GR) metrics were calculated according to Hafner et al. 2016 [19]. GR50 curves were visualized using the GR-calculator webserver (www.grcalculator.org) [29].

### 3.9. Molecular Modelling

The Protein Preparation Wizard in Maestro Software version 11.1 was used to prepare the proteins structures [30]. The cytochrome P450 17A1 structures were obtained from Protein Data Bank [31] (PDB protein codes 3SWZ [10] and 5IRQ [11]). Bond orders were assigned, hydrogens were added, and zero-order bonds to metals were created. For protein structures the A chains were selected, and all water molecules were removed. The formal charge on heme iron was set to +3 and non-protonated ligand state was used. The hydrogen bonding network was optimized at pH 7.0. A restrained protein minimization was performed using OPLS3 [32] force field with convergence of heavy atoms to RMSD 0.30 Å. Ligands preparation was performed with LigPrep in Maestro [30]. Possible tautomers and protonation states were generated at pH 7.0 ± 2.0. The Epik program was used to predict pKa values of ligands [33]. Docking was performed with GOLD (Genetic Optimisation for Ligand Docking) program version 5.6 [34]. Proteins prepared by Protein Preparation Wizard were used without additional modifications in GOLD. The co-crystalized ligand was extracted, and the binding site was defined around the center of the mass of the co-crystalized ligand within 15 Å. Ligands prepared by LigPrep were exported from Maestro. Ligands were docked 10 times with slow genetic algorithm and with ChemScore as the scoring function [35]. For constrained docking the distance between the heme iron and the atom expected to be coordinated to Fe was constrained between 1.5 and 3.5 Å.

The Desmond system builder was used to create the molecular dynamics (MD) systems with the protein-ligand complex embedded in a SPC water model yielding an orthorhombic box with a buffer size of 10 Å between the protein and the box boundary. The final system comprised close to 70,000 atoms including approximately 7500 atoms for the protein including the heme group, 36 atoms for the ligand (in the case of **1a**), one chloride ion to neutralize the system, and approximately 21,000 water molecules. The MD simulations were performed with the Desmond program (version 3.6) using the OPLS-2005 force field [36]. For equilibration of the system prior to the production runs, the Desmond default equilibration protocol was used. Subsequently, the systems were simulated for 100 ns and 1000 frames collected. A total of 36 simulations were performed based on the 3SWZ and 5IRQ protein structures combined with the different poses from the GOLD dockings. For each of the MD simulations the free energy of binding were determined by the Molecular Mechanics/Generalized Born Surface Area (MM/GBSA) method [37] using the thermal_mmgbsa.py script provided as part of the Schrodinger software system [38]. The values listed in Table 2 are averages of 4–6 determinations each based on a 100 ns MD simulation.

## 4. Conclusions

In summary, a set of early hit molecules based on a benzimidazole/indole scaffold were found to have sub-micromolar inhibitory activity for CYP17A1 with two most potent molecules being **1e** IC_50_ = 0.56 µM and **1c** IC_50_ = 0.83 µM. Our compounds incorporate a novel, non-steroidal scaffold and, due to a simple chemistry, allow for the rapid generation of a library of easily accessible analogues.

Compound **1c** displayed favourable inhibitory selectivity against other drug-metabolizing CYP family enzymes, however it demonstrated only two-fold selectivity for CYP17A1 over CYP21A2 inhibition. Furthermore, compound **1c** displayed high cytotoxicity towards PC3 cancer cells while maintaining remarkably non-toxicity towards fibroblasts. This constitutes a starting point for further optimization towards small molecule therapeutics for the treatment of CRPC.

## Figures and Tables

**Table 1:** Binding and enzyme inhibition; ND—not determined; *—data from Ref. [10], **—data from Ref. [11].

Compound	CYP17A1 Kd (nM)	CYP17A1 IC_50_ (µM)	CYP21A2 IC_50_ (µM)	CYP3A4 IC_50_ (µM)	CYP2D6 IC_50_ (µM)
**1a**	290 ± 55	8.06 ± 3.9	ND	ND	ND
**1b**	420 ± 70	>10	ND	ND	ND
**1c**	96 ± 22	0.83 ± 0.19	1.5 ± 0.67	>10	>10
**1d**	150 ± 37	1.76 ± 0.19	ND	ND	ND
**1e**	120 ± 34	0.56 ± 0.10	0.19 ± 0.03	>10	2.5 ± 0.60
**Abiraterone**	<100 *	0.08 **	-	-	-
**Galeterone**	<100 *	0.13 **	-	-	-
**Orteronel**	<40 **	0.95 **	-	-	-

**Table 2:** Average binding free energy of compounds **1a**-**1e** with two possible binding modes. n/a—non applicable.

Compound	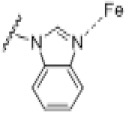 Kcal/mol	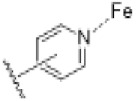 Kcal/mol
**1a**	–18.7	–24.6
**1b**	–20.8	n/a
**1c**	–18.5	–26.7
**1d**	–19.9	n/a
**1e**	n/a	–23.0

## Supplementary Materials

Supplementary materials can be found at https://www.mdpi.com/1422-0067/21/14/4868/s1.

## Abbreviations

PCa	prostate cancer
ADT	androgen deprivation therapy
CRPC	castration resistant prostate cancer
AR	androgen receptor
PSA	prostate specific antigen
hK1	human glandular kallikrein 1
MD	molecular dynamics
ABT	abiraterone
5-FU	5-fluorouracil
GnRH	gonadotropin releasing hormone
