# Supplementary material for: Discovery of Novel Non-Steroidal Cytochrome P450 17A1 Inhibitors as Potential Prostate Cancer Agents
Source: Int J Mol Sci. 2020 Jul 9;21(14):4868. doi: 10.3390/ijms21144868 (PMC7402352; doi:10.3390/ijms21144868)
Supplement: Supplementary file 1 [file ijms-21-04868-s001.pdf]

Supplementary Information

Contents

S1. Chemistry with analytical data 2

S2. Binding spectra and CYP IC_{50} curves 20

S3. Compound dose-response (GR_{50}) in PC-3 cells 22

S4. Docking poses 24

S1. Chemistry with analytical data

1-(4-bromophenyl)-1*H*-benzo[*d*]imidazole (2a)

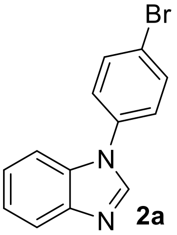

2a

Benzimidazole (5.91 g, 50 mmol), 1-bromo-4-fluorobenzene (9.63 g, 55 mmol), and potassium phosphate (21.23 g, 100 mmol) were stirred in anhydrous DMF (200 mL) overnight at 160 °C. Reaction was partitioned between 100 mL of DCM and 400 mL of water. Aqueous was extracted twice with 100 mL of DCM, combined organics were washed three times with 100 mL of water and dried with anhydrous $MgSO_4$. Crude was purified by column chromatography eluting with heptane / EtOAc (50:50) and recrystallized from EtOAc / heptane (1:1) to obtain the title compound as a white solid (10.6 g, 78%).

^{1}H NMR (600 MHz, DMSO-d_6) δ 8.58 (s, 1H), 7.85 – 7.77 (m, 3H), 7.70 – 7.65 (m, 2H), 7.65 – 7.61 (m, 1H), 7.34 (pd, *J* = 7.2, 1.4 Hz, 2H). ^{13}C NMR (151 MHz, DMSO-d_6) δ 143.8, 143.1, 135.2, 132.9, 132.9, 132.9, 132.8, 132.8, 125.7, 125.6, 123.5, 122.5, 120.2, 119.9, 110.5.

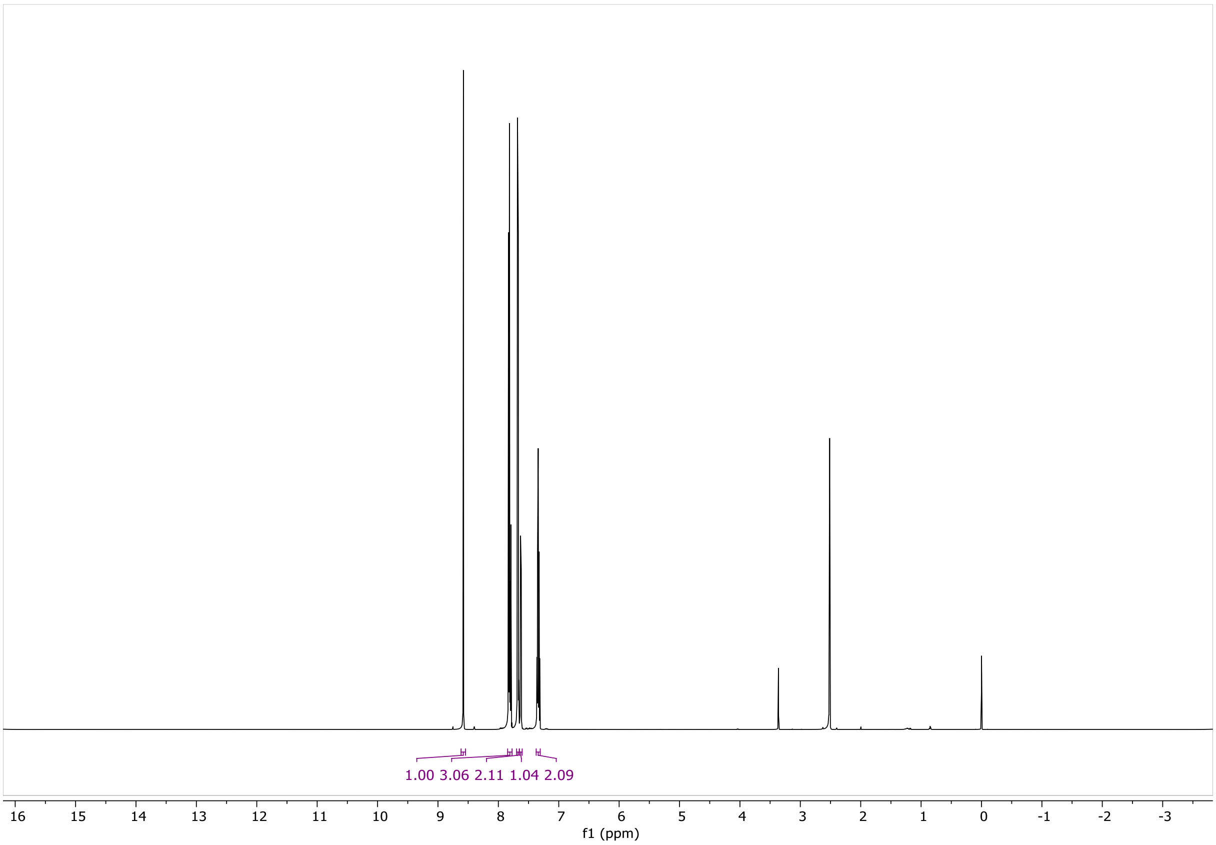

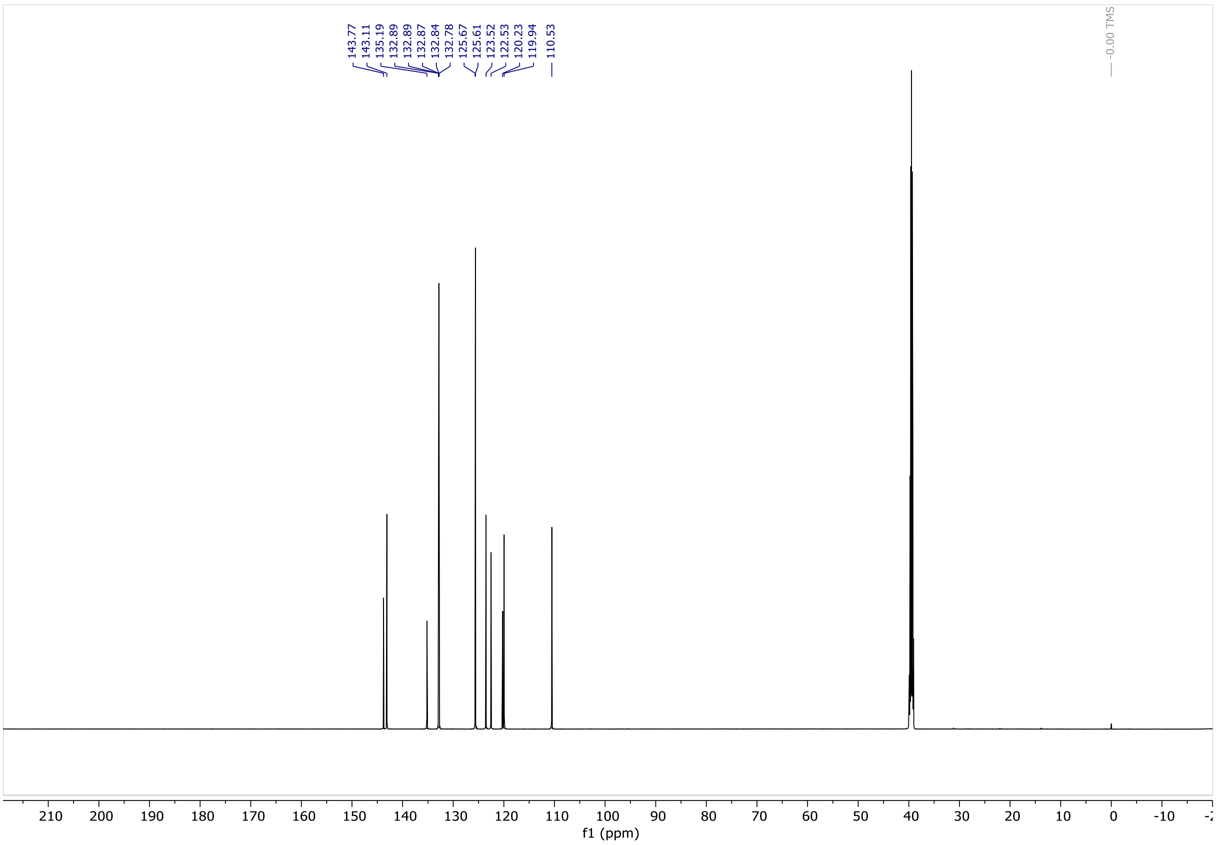
143.77
143.11
135.19
132.89
132.89
132.87
132.84
132.78
125.67
125.61
123.52
122.53
120.23
119.94
110.53
-0.00 TMS
f1 (ppm)

1-(4-bromophenyl)-1H-indole (2b)

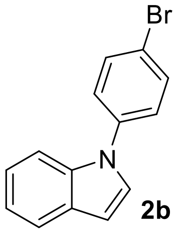

2b

Indole (1.76 g, 15 mmol), 1-bromo-4-fluorobenzene (5.25 g, 30 mmol), and potassium phosphate (15.92 g, 75 mmol) were stirred in anhydrous DMF (150 mL) overnight at 150 °C. Reaction was diluted with 200 mL of ether, washed 3 × 150 mL of water and dried with anhydrous $MgSO_4$. Crude was purified by column chromatography eluting with heptane / EtOAc (95:5) to obtain the title compound as a colourless oil (2.15 g, 53%).

^{1}H NMR (600 MHz, $CDCl_3$) δ 7.72 (dt, *J* = 7.8, 1.0 Hz, 1H), 7.67 – 7.64 (m, 2H), 7.55 (dt, *J* = 8.3, 0.9 Hz, 1H), 7.42 – 7.39 (m, 2H), 7.31 (d, *J* = 3.3 Hz, 1H), 7.26 (ddd, *J* = 8.3, 6.1, 1.3 Hz, 1H), 7.21 (ddd, *J* = 8.0, 7.1, 1.1 Hz, 1H), 6.72 (dd, *J* = 3.3, 0.9 Hz, 1H). ^{13}C NMR (151 MHz, $CDCl_3$) δ 139.0, 135.8, 132.9, 129.5, 127.7, 125.9, 122.8, 121.4, 120.8, 119.8, 110.4, 104.3.

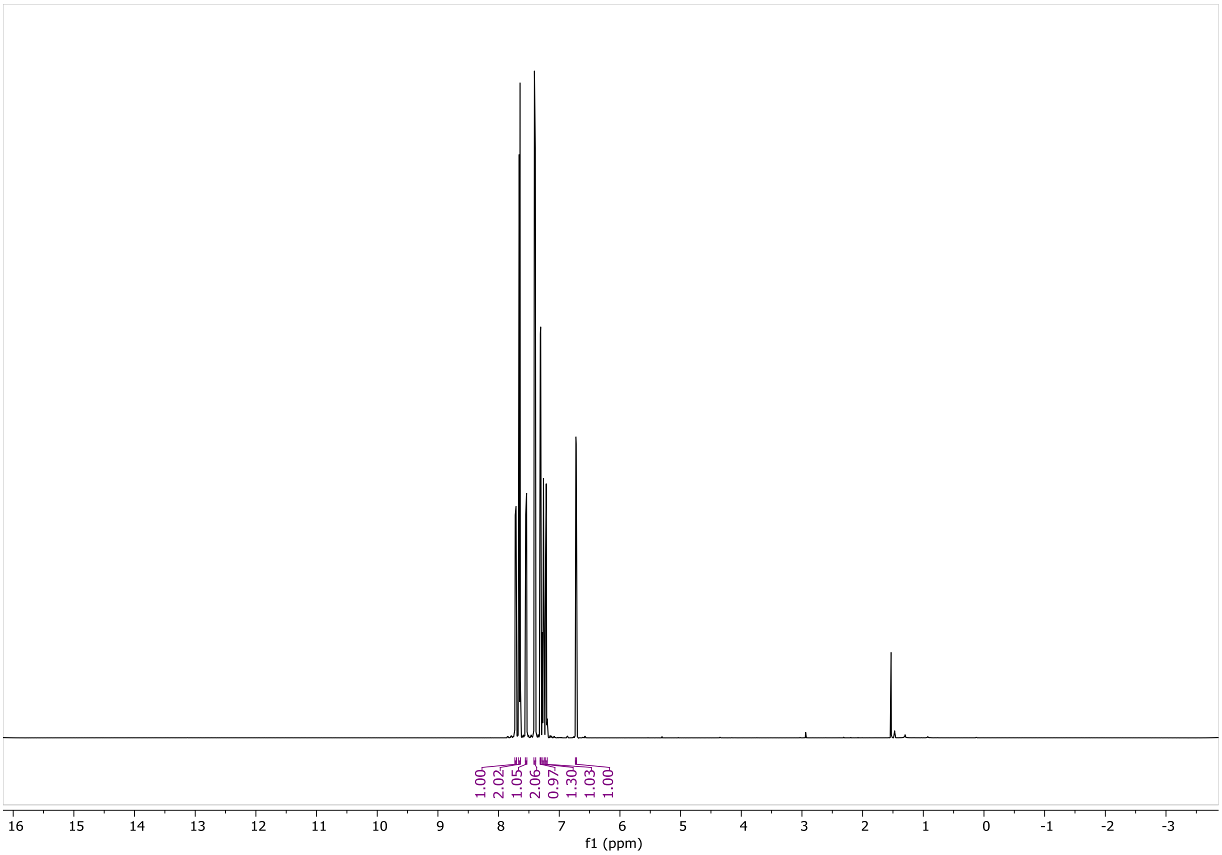

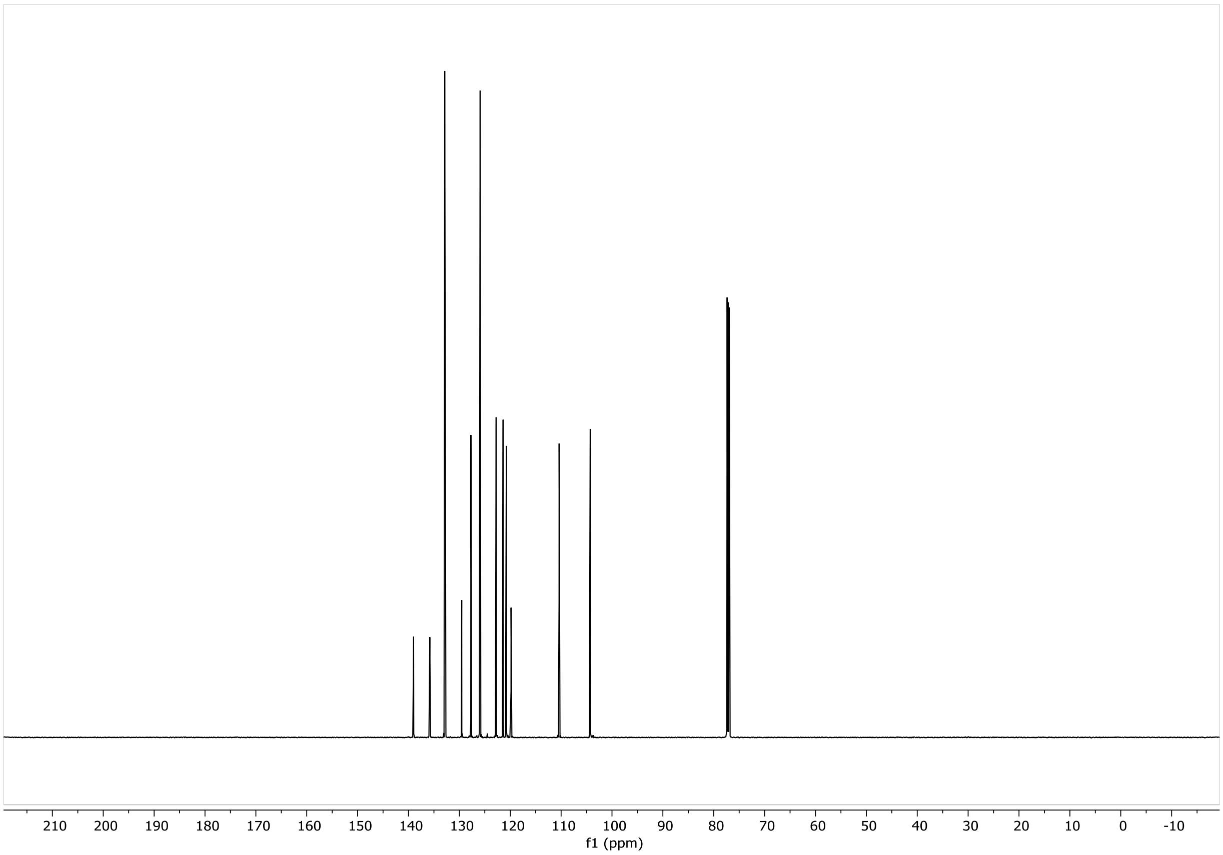
210 200 190 180 170 160 150 140 130 120 110 100 90 80 70 60 50 40 30 20 10 0 -10
f1 (ppm)

1-(4-nitrophenyl)-1*H*-benzo[d]imidazole (2c)

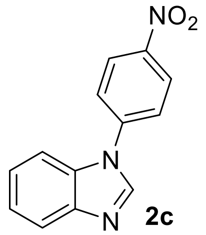

2c

Benzimidazole (5.91 g, 50 mmol), 1-fluoro-4-nitrobenzene (7.76 g, 55 mmol), and potassium phosphate (21.23 g, 100 mmol) were stirred in anhydrous DMF (200 mL) overnight at 150 °C. Reaction was diluted with 500 mL of water and extracted 3 × 200 mL of ether. Combined organics were washed with brine and dried with anhydrous $MgSO_4$. Crude was purified by dissolving in hot EtOH, concentrating on a rotavap, and filtering the formed solid. Thus, the title compound was obtained as a light brown solid (7.94 g, 66%).

^{1}H NMR (400 MHz, DMSO-*d*$_6$) δ 8.73 (s, 1H), 8.49 – 8.39 (m, 2H), 8.07 – 7.98 (m, 2H), 7.85 – 7.72 (m, 2H), 7.37 (pd, *J* = 7.2, 1.4 Hz, 2H). ^{13}C NMR (101 MHz, DMSO-*d*$_6$) δ 145.7, 144.1, 143.2, 141.3, 132.3, 125.5, 124.0, 123.7, 123.2, 120.2, 111.0.

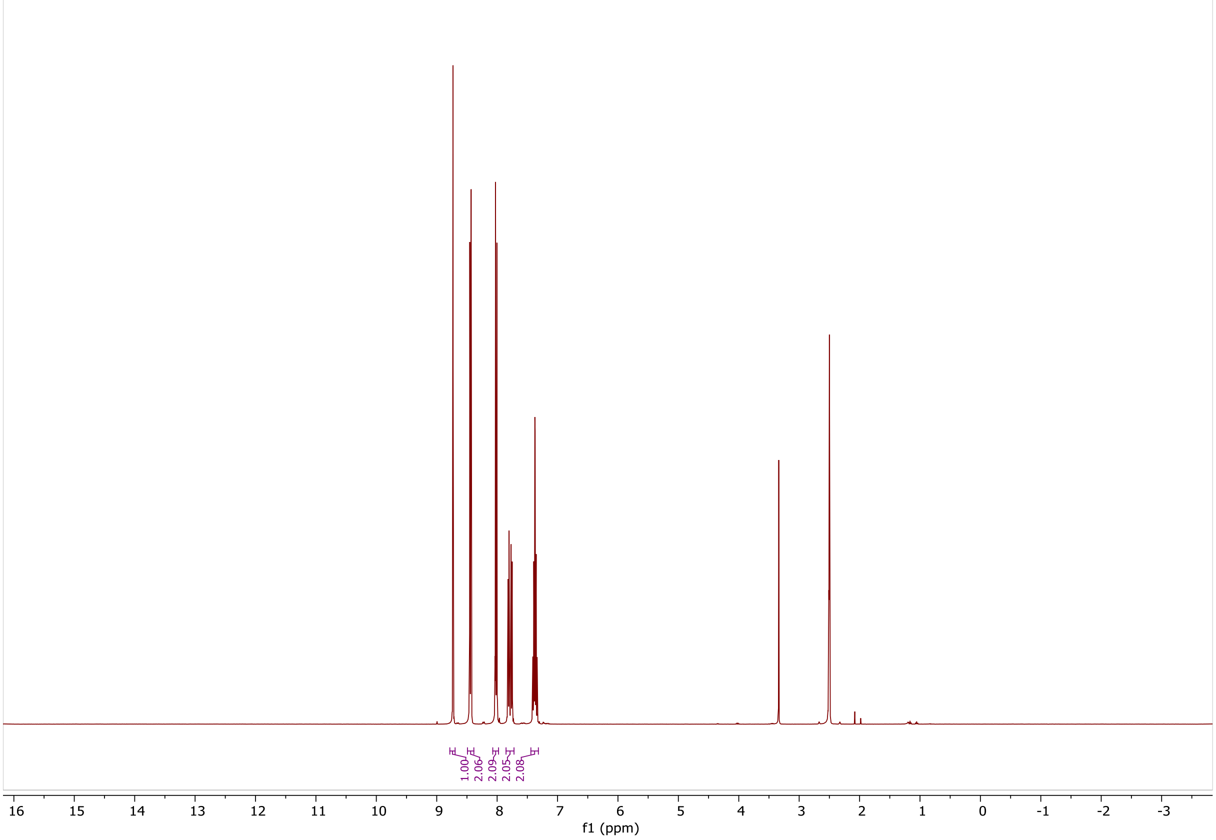

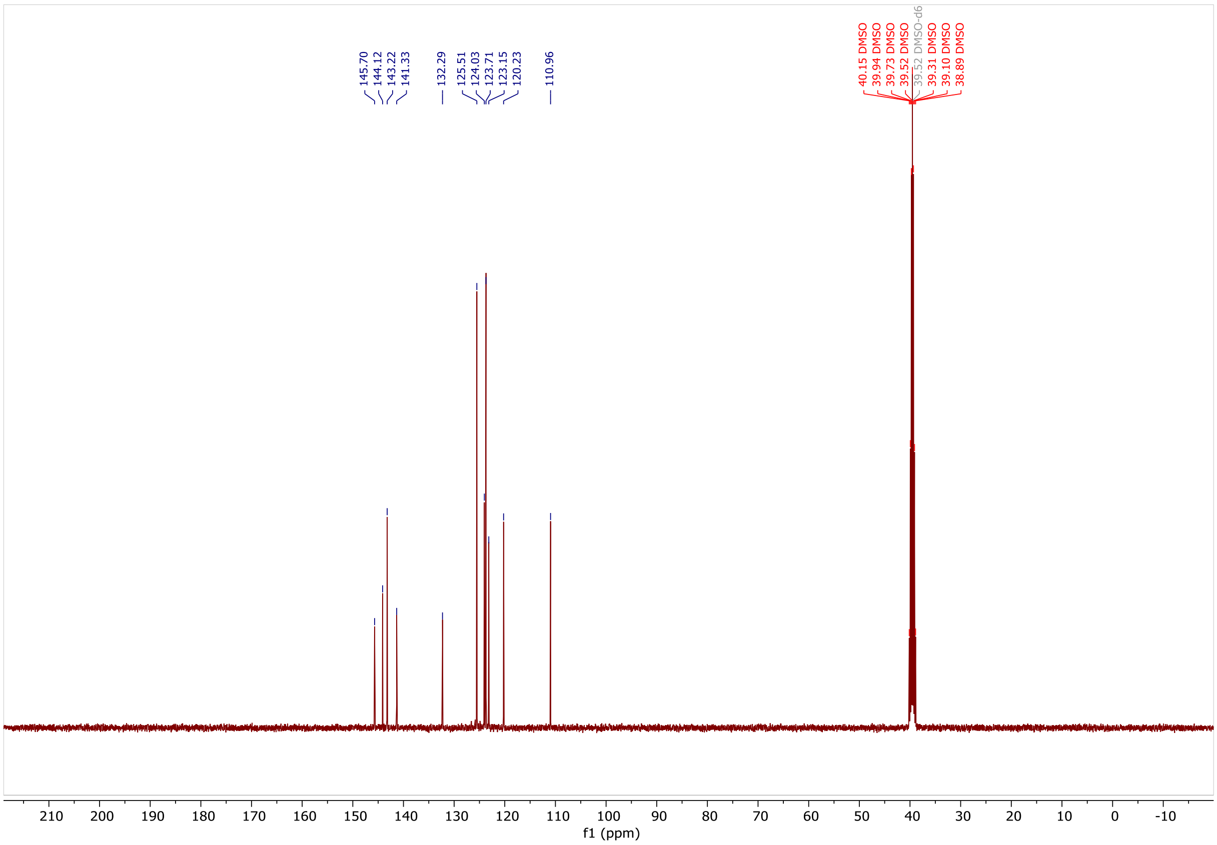
145.70
144.12
143.22
141.33
132.29
125.51
124.03
123.71
123.15
120.23
110.96
40.15 DMSO
39.94 DMSO
39.73 DMSO
39.52 DMSO
39.52 DMSO-d6
39.31 DMSO
39.10 DMSO
38.89 DMSO
210 200 190 180 170 160 150 140 130 120 110 100 90 80 70 60 50 40 30 20 10 0 -10
f1 (ppm)

4-(1*H*-benzo[d]imidazol-1-yl)aniline (2d)

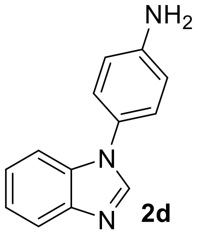

2d

Benzimidazole derivative **2c** (4.68 g, 19.6 mmol) was dissolved in 100 mL of MeOH and hydrogenated at room temperature and pressure overnight using 208 mg (0.2 mmol, 1mol%) of 10% Pd/C. Reaction was filtered from the catalyst and solvent removed on a rotavap. Crude was purified by column chromatography on silica eluting with 2M NH_3 MeOH/EtOAc (5:95) to obtain the title compound as an off-white oil (3.37 g, 82%). This compound can be converted into HCl salt and recrystallized from water/EtOH to produce spectacularly long, fine needles. NMR data below pertains to HCl salt.

^{1}H NMR (600 MHz, D_2O) δ 9.45 (s, 1H), 7.82 (dt, *J* = 8.3, 0.8 Hz, 1H), 7.80 – 7.76 (m, 2H), 7.65 (dd, *J* = 8.4, 1.7 Hz, 3H), 7.62 – 7.54 (m, 2H). ^{13}C NMR (151 MHz, D_2O) δ 140.0, 133.2, 132.9, 131.1, 130.4, 127.4, 127.4, 126.8, 124.9, 114.9, 112.9.

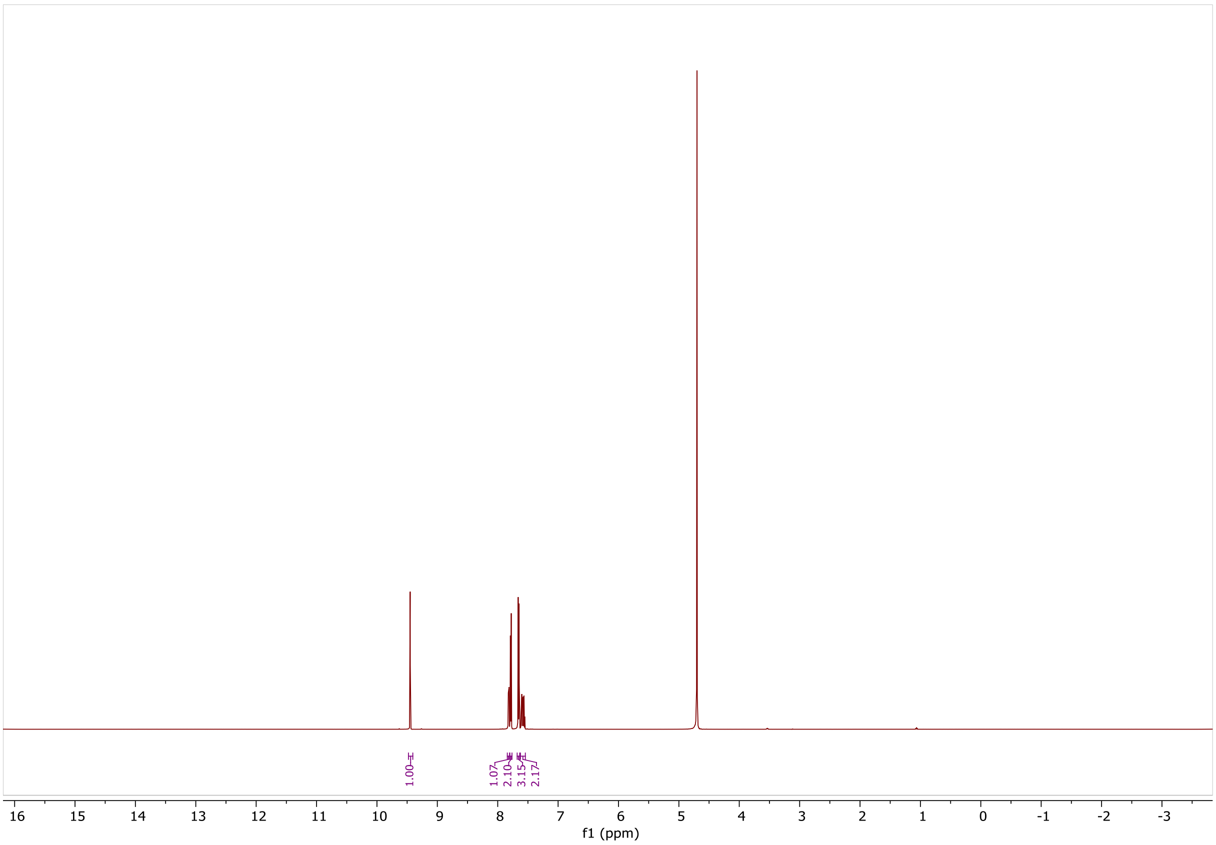

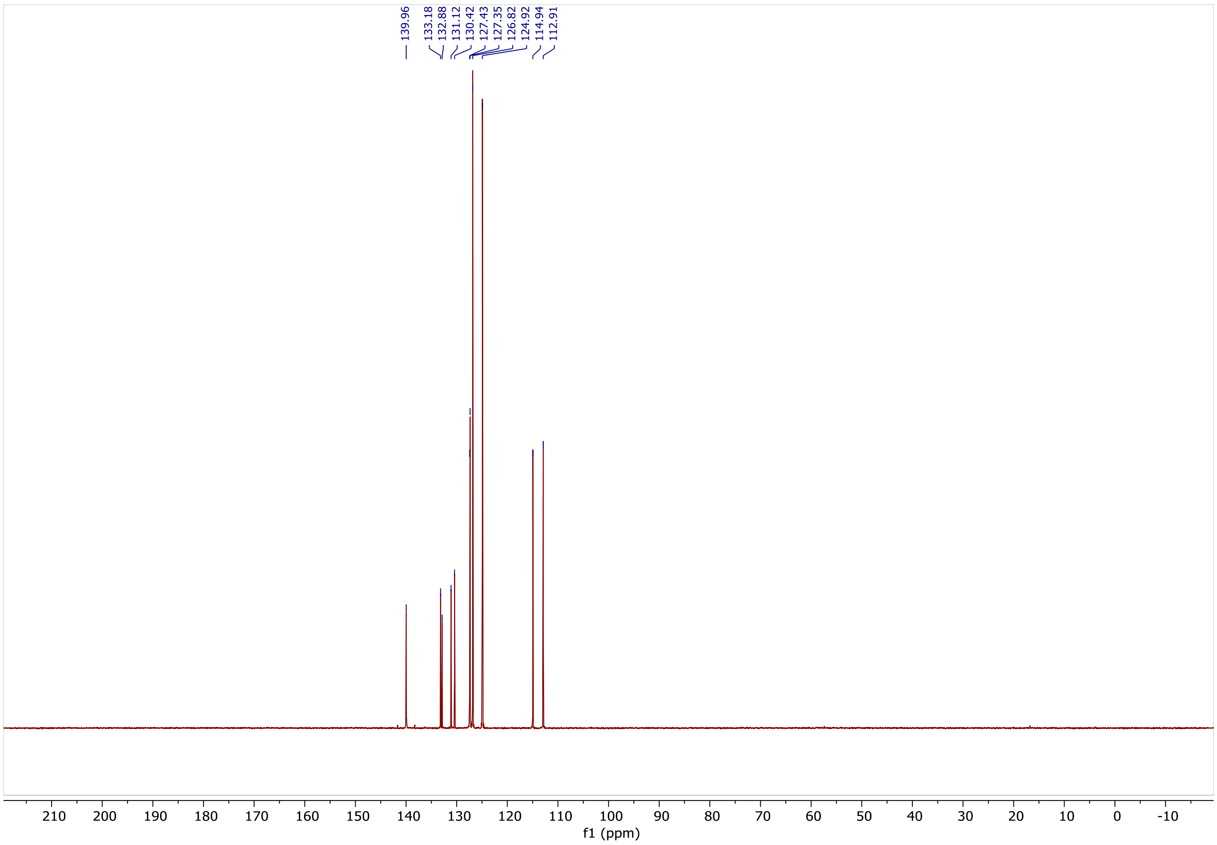
139.96
133.18
132.88
131.12
130.42
127.43
127.35
126.82
124.92
114.94
112.91
210 200 190 180 170 160 150 140 130 120 110 100 90 80 70 60 50 40 30 20 10 0 -10
f1 (ppm)

N-(4-(1H-benzo[d]imidazol-1-yl)phenyl)pyridin-4-amine (1a)

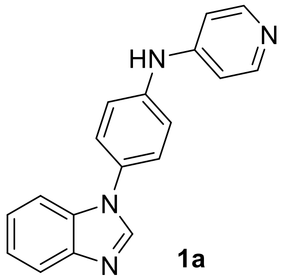

1a

A flame-dried vial was charged with **2a** (137 mg, 0.5 mmol), 4-aminopyridine (57 mg, 0.6 mmol), tBuXPhos Pd G1 (17 mg, 0.025 mmol), tBuXPhos (11 mg, 0.025 mmol), and sodium t-butoxide (96 mg, 1 mmol). It was sealed and evacuated/backfilled with argon three times. 2 mL of anhydrous THF was added and the reaction was stirred overnight at room temperature. It was diluted with 5 mL of DMF and processed through SCX column. Crude was recrystallized from MeOH to obtain the title compound as an off-white solid (51 mg, 36%).

^{1}H NMR (600 MHz, DMSO-d_6) δ 9.05 (s, 1H), 8.51 (s, 1H), 8.31 – 8.19 (m, 2H), 7.81 – 7.76 (m, 1H), 7.66 – 7.62 (m, 2H), 7.61 – 7.58 (m, 1H), 7.46 – 7.40 (m, 2H), 7.36 – 7.29 (m, 2H), 7.04 – 6.97 (m, 2H). ^{13}C NMR (151 MHz, DMSO-d_6) δ 150.2, 149.5, 143.6, 143.2, 140.2, 133.3, 129.9, 124.9, 123.2, 122.2, 120.5, 119.8, 110.5, 109.5. HRMS (MALDI): *m/z* calculated for $[M+H]^+$ 287.12912, found 287.12911.

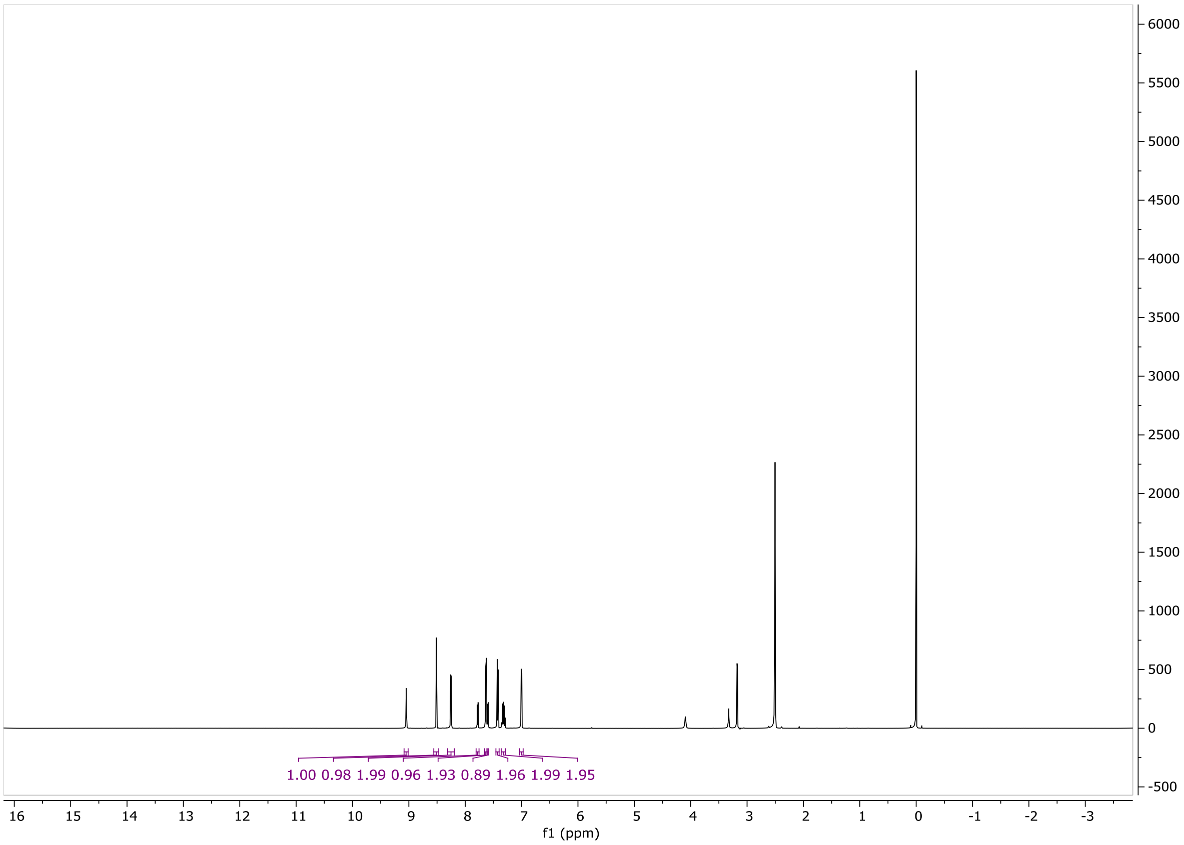

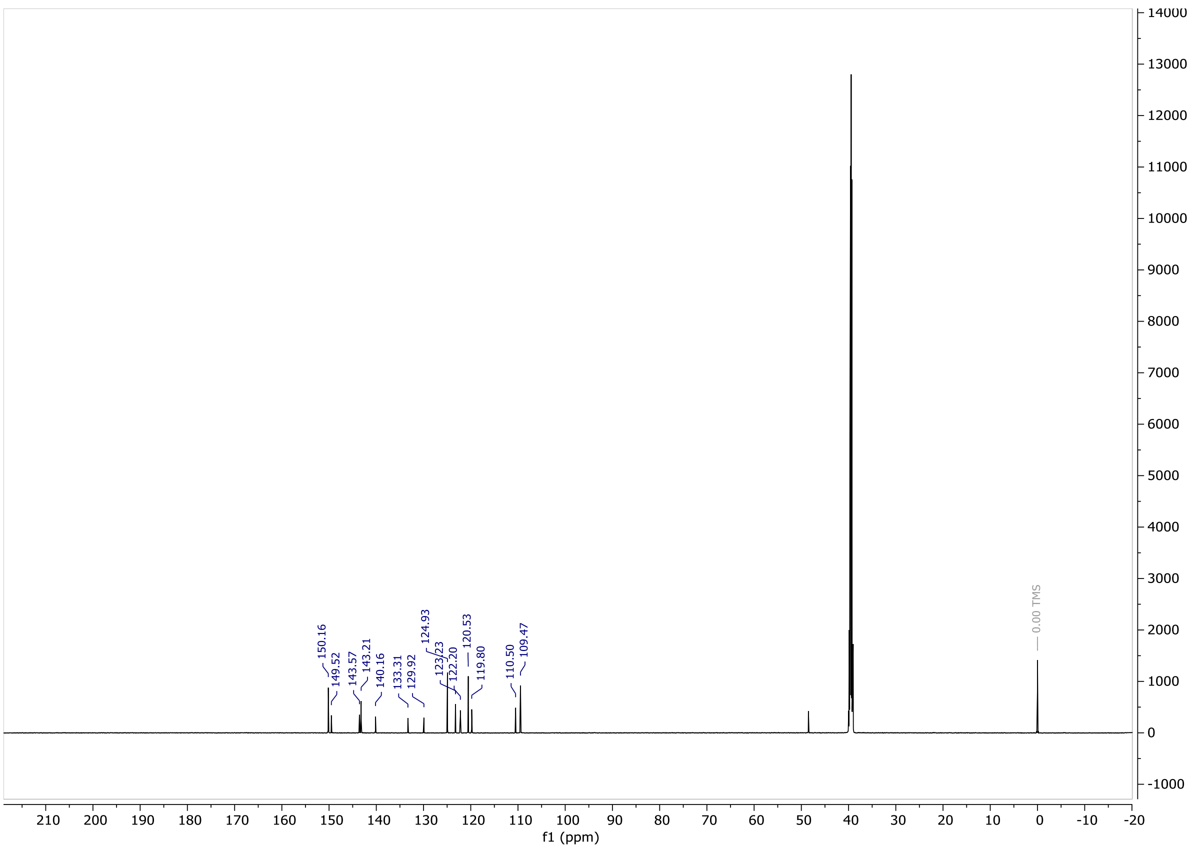

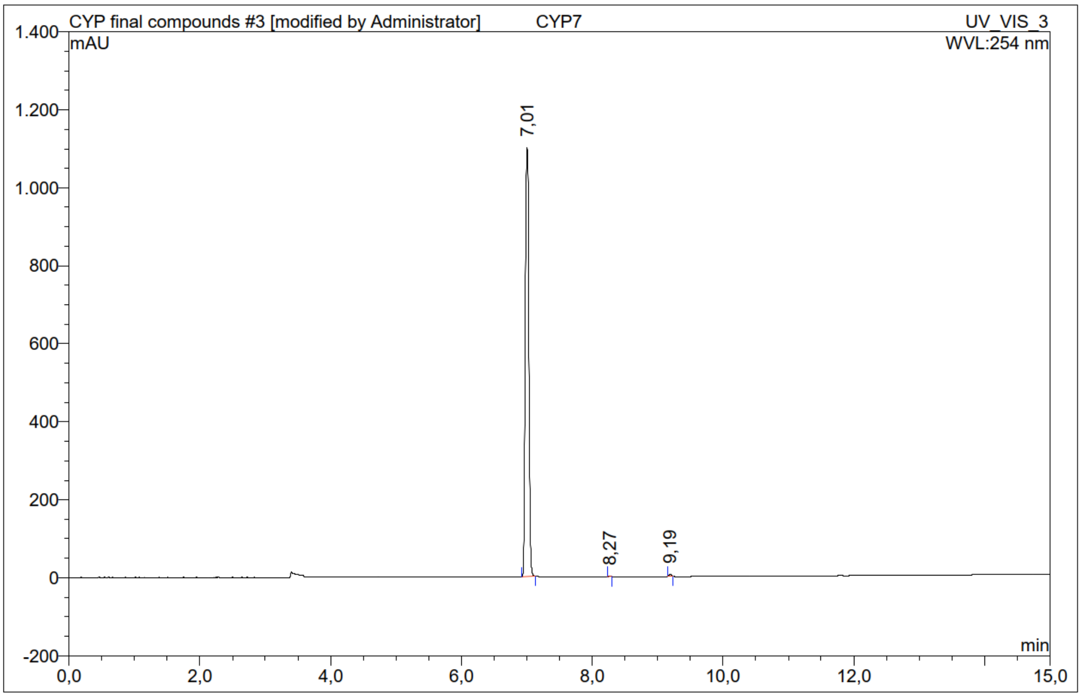

No.	Ret.Time min	Peak Name	Height mAU	Area mAU*min	Rel.Area %	Amount	Resolution(EP)
1	7,01	n.a.	1100,800	67,125	99,58	n.a.	15,13
2	8,27	n.a.	1,448	0,057	0,09	n.a.	13,43
3	9,19	n.a.	5,305	0,225	0,33	n.a.	n.a.
Total:			1107,552	67,407	100,00	0,000	

N-(4-(1H-benzo[d]imidazol-1-yl)phenyl)pyridin-2-amine (1b)

A flame-dried vial was charged with **2a** (137 mg, 0.5 mmol), 2-aminopyridine (57 mg, 0.6 mmol), tBuXPhos Pd G1 (17 mg, 0.025 mmol), tBuXPhos (11 mg, 0.025 mmol), and sodium t-butoxide (96 mg, 1 mmol). It was sealed and evacuated/backfilled with argon three times. Subsequently, 3 mL of anhydrous DMF was added and the reaction was stirred overnight at room temperature. Then, 0.3 mL of water was added and the reaction was processed through SCX column. Crude was purified by preparative HPLC and freeze dried as a HCl salt to obtain the title compound as a white solid (20 mg, 14%). NMR data below pertains to HCl salt.

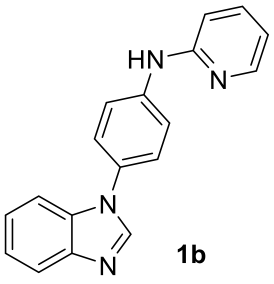

1b

^{1}H NMR (600 MHz, D_2O) δ 9.52 (s, 1H), 8.17 – 8.11 (m, 1H), 8.02 – 7.96 (m, 2H), 7.89 (d, *J* = 8.7 Hz, 2H), 7.85 (d, *J* = 8.0 Hz, 1H), 7.77 – 7.70 (m, 4H), 7.38 (d, *J* = 9.1 Hz, 1H), 7.18 (t, *J* = 7.0 Hz, 1H). ^{13}C NMR (151 MHz, D_2O) δ 151.2, 145.4, 140.1, 137.5, 136.1, 131.7, 131.5, 131.1, 127.2, 127.2, 126.9, 125.7, 115.3, 115.2, 113.9, 113.0. HRMS (MALDI): *m/z* calculated for $[M+H]^+$ 287.12912, found 287.12907.

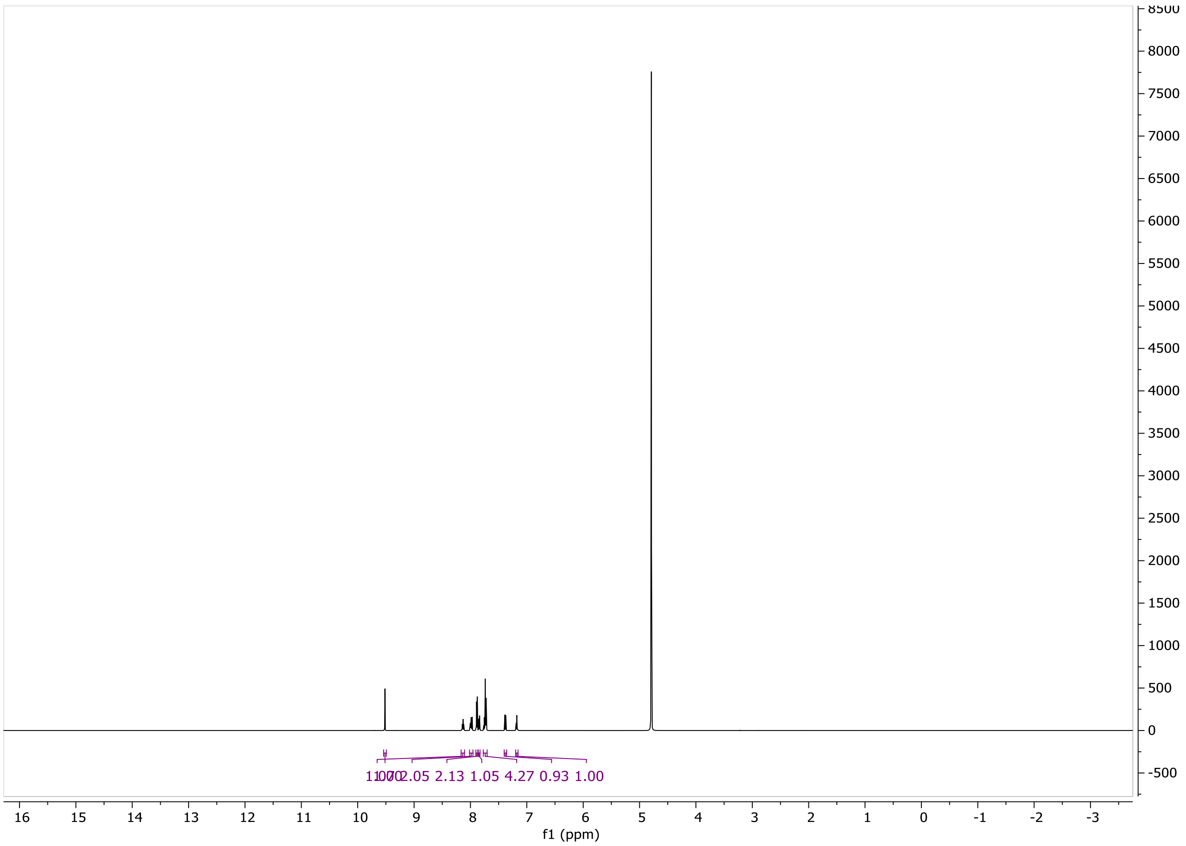

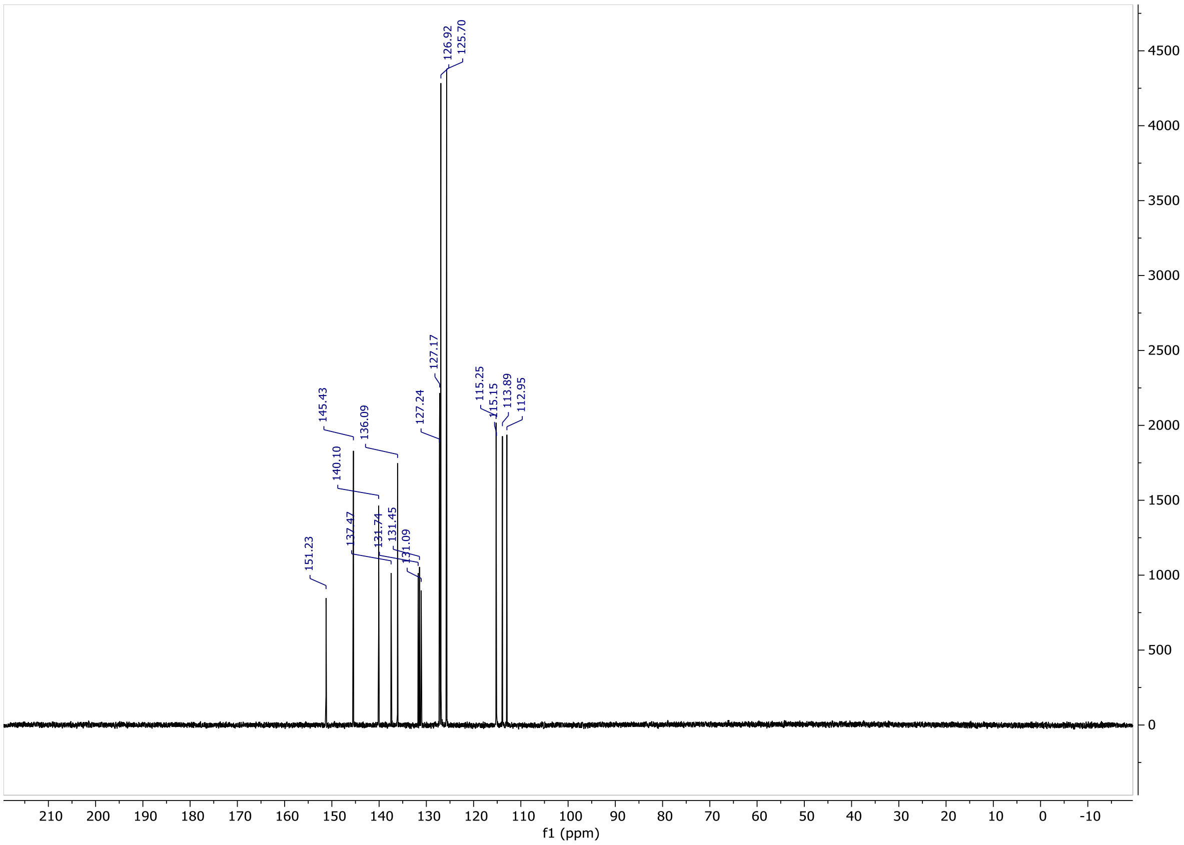

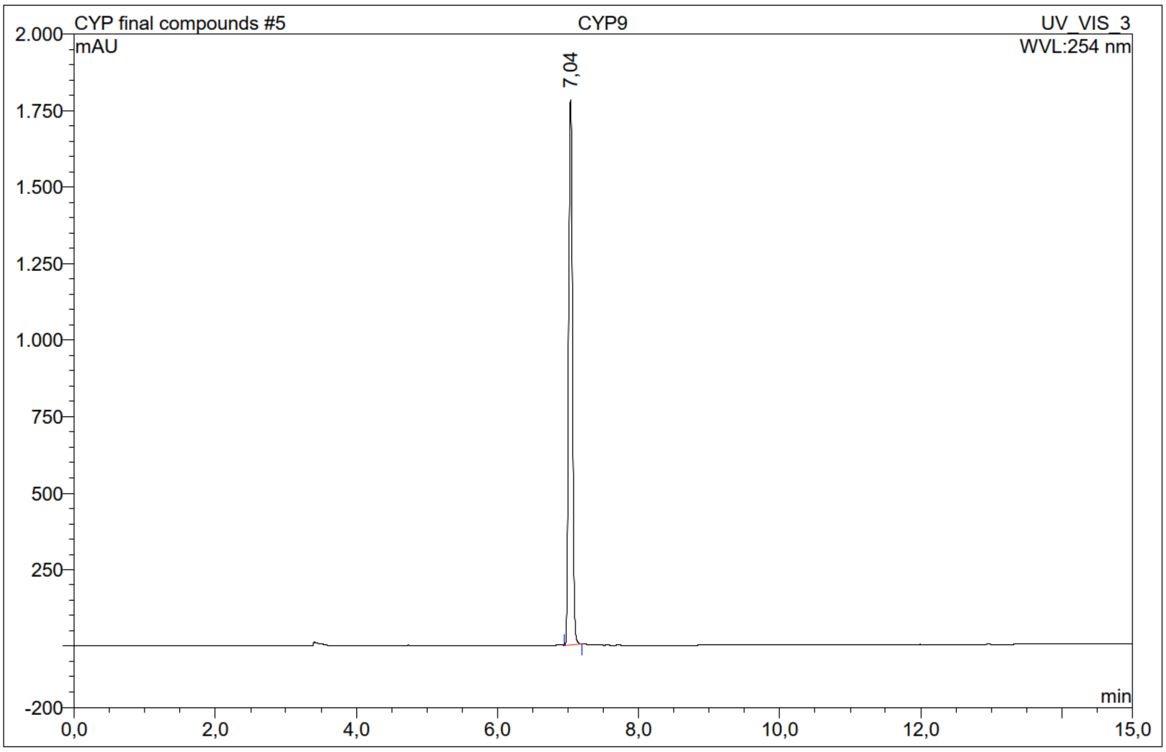

No.	Ret.Time min	Peak Name	Height mAU	Area mAU*min	Rel.Area %	Amount	Resolution(EP)
1	7,04	n.a.	1782,332	112,956	100,00	n.a.	n.a.
Total:			1782,332	112,956	100,00	0,000	

N-(4-(1H-benzo[d]imidazol-1-yl)phenyl)pyridin-3-amine (1c)

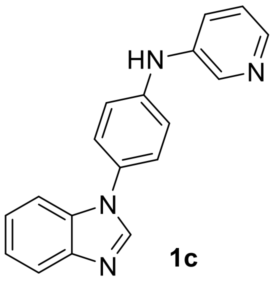

1c

A flame-dried vial was charged with 3-bromopyridine (158 mg, 1 mmol), **2d** (251 mg, 1.2 mmol), tBuXPhos Pd G3 (16 mg, 0.02 mmol), tBuXPhos (8 mg, 0.01 mmol), and sodium t-butoxide (192 mg, 2 mmol). The vial was sealed and evacuated/backfilled with argon three times. Then, 5 mL of anhydrous THF was added. Reaction was stirred at 100 °C overnight. It was then diluted with 25 mL of EtOAc and filtered through silica. After solvent removal, remaining residue was passed through silica eluting 2M NH_3 in MeOH / DCM (2:98 → 5:95), purified by preparative HPLC, and processed through SCX column to obtain the title compound as an off-white solid after trituration with ether (34 mg, 12%).

^{1}H NMR (600 MHz, DMSO-d_6) δ 8.65 (s, 1H), 8.46 (s, 1H), 8.44 (dd, J = 2.8, 0.7 Hz, 1H), 8.11 (dd, J = 4.6, 1.4 Hz, 1H), 7.79 – 7.74 (m, 1H), 7.60 – 7.52 (m, 4H), 7.35 – 7.27 (m, 5H). ^{13}C NMR (151 MHz, DMSO-d_6) δ 143.5, 143.2, 142.4, 141.2, 139.7, 139.3, 133.5, 128.1, 125.1, 123.8, 123.1, 123.1, 122.1, 119.7, 117.4, 110.5. HRMS (MALDI): m/z calculated for $[M+H]^+$ 287.12912, found 287.12939.

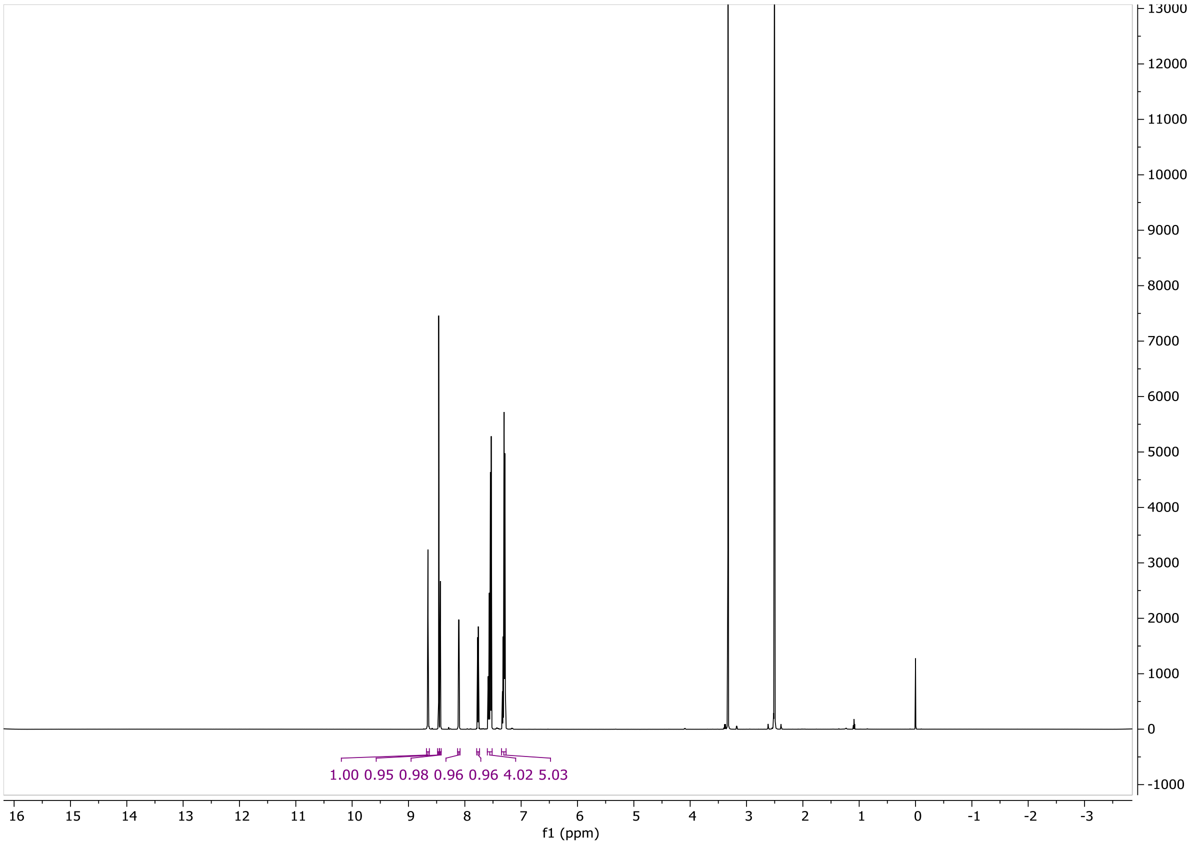

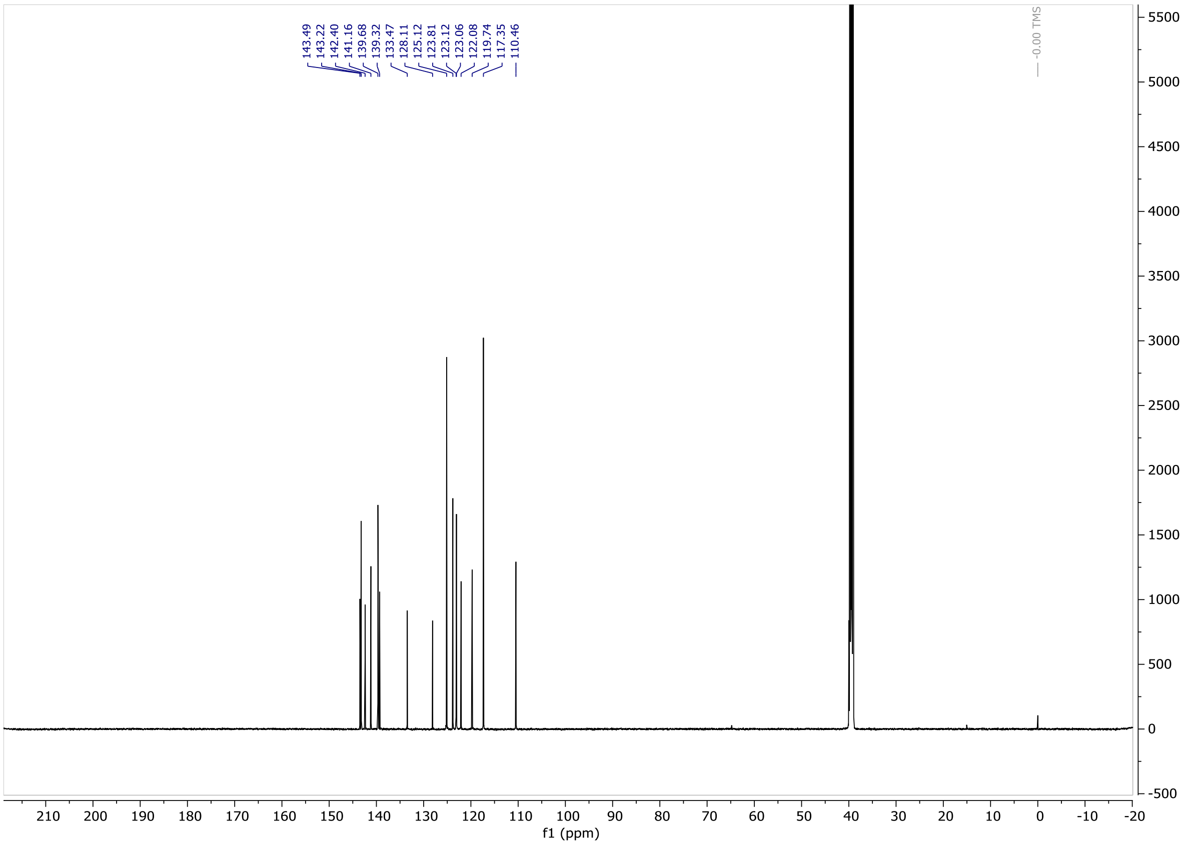

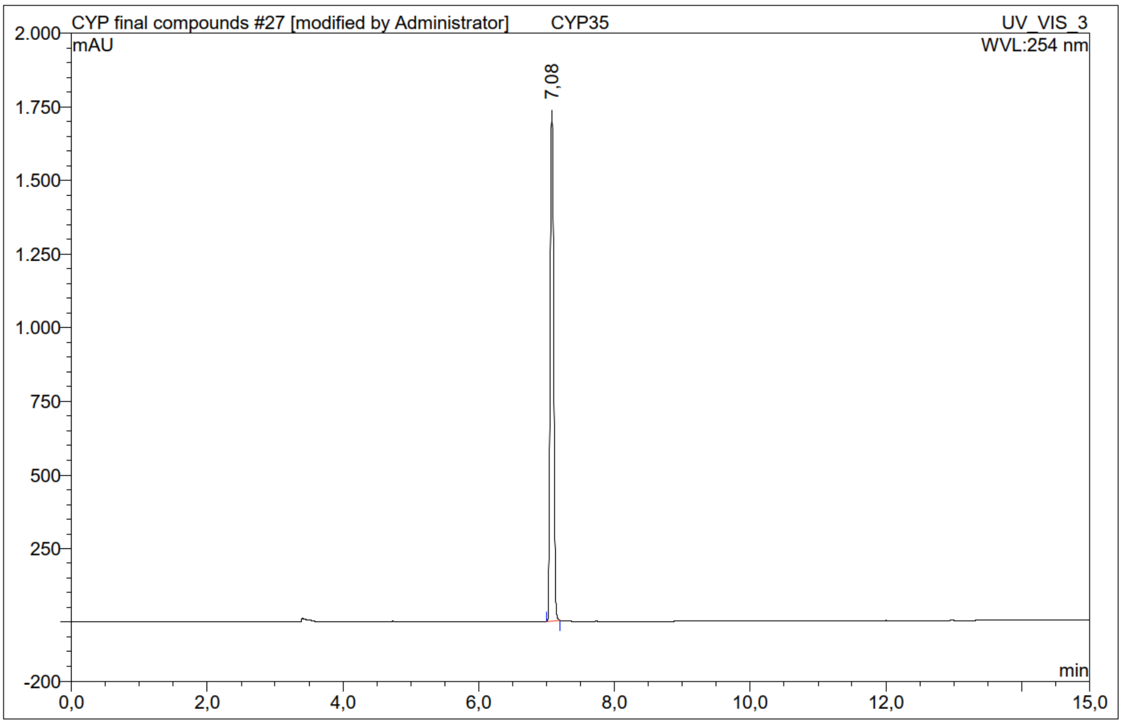

No.	Ret.Time min	Peak Name	Height mAU	Area mAU*min	Rel.Area %	Amount	Resolution(EP)
1	7,08	n.a.	1735,189	102,487	100,00	n.a.	n.a.
Total:			1735,189	102,487	100,00	0,000	

4-(1*H*-benzo[*d*]imidazol-1-yl)-*N*-phenylaniline (1d)

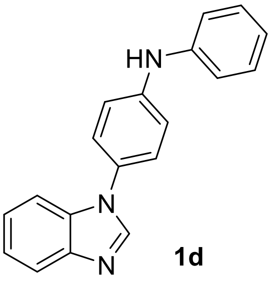

1d

A flame-dried vial was charged with **2a** (273 mg, 1 mmol), tBuXPhos Pd G1 (7 mg, 0.01 mmol), tBuXPhos (4 mg, 0.01 mmol) and sodium t-butoxide (115 mg, 1.2 mmol). It was sealed and evacuated/backfilled with argon three times. Aniline (0.11 mL, 1.2 mmol, freshly distilled) was added followed by 2 mL of anhydrous THF. Reaction was stirred at 60 °C for 2 hours. It was then diluted with 10 mL of EtOAc and filtered through Celite. Crude was purified by column chromatography on silica eluting with EtOAc/heptane (40:60 → 100:0) to obtain the title compound as an off-white solid (184 mg, 65%).

^{1}H NMR (600 MHz, DMSO-d_6) δ 8.48 (s, 1H), 8.45 (s, 1H), 7.79 – 7.75 (m, 1H), 7.57 – 7.53 (m, 1H), 7.49 (d, *J* = 8.7 Hz, 2H), 7.34 – 7.25 (m, 6H), 7.18 (d, *J* = 8.6 Hz, 2H), 6.94 – 6.88 (m, 1H). ^{13}C NMR (151 MHz, DMSO-d_6) δ 143.5, 143.4, 143.2, 142.7, 133.6, 129.2, 127.3, 125.0, 123.1, 122.0, 120.4, 119.7, 117.5, 116.8, 110.5. HRMS (MALDI): *m*/*z* calculated for [M+H]$^+$ 286.13387, found 286.13384.

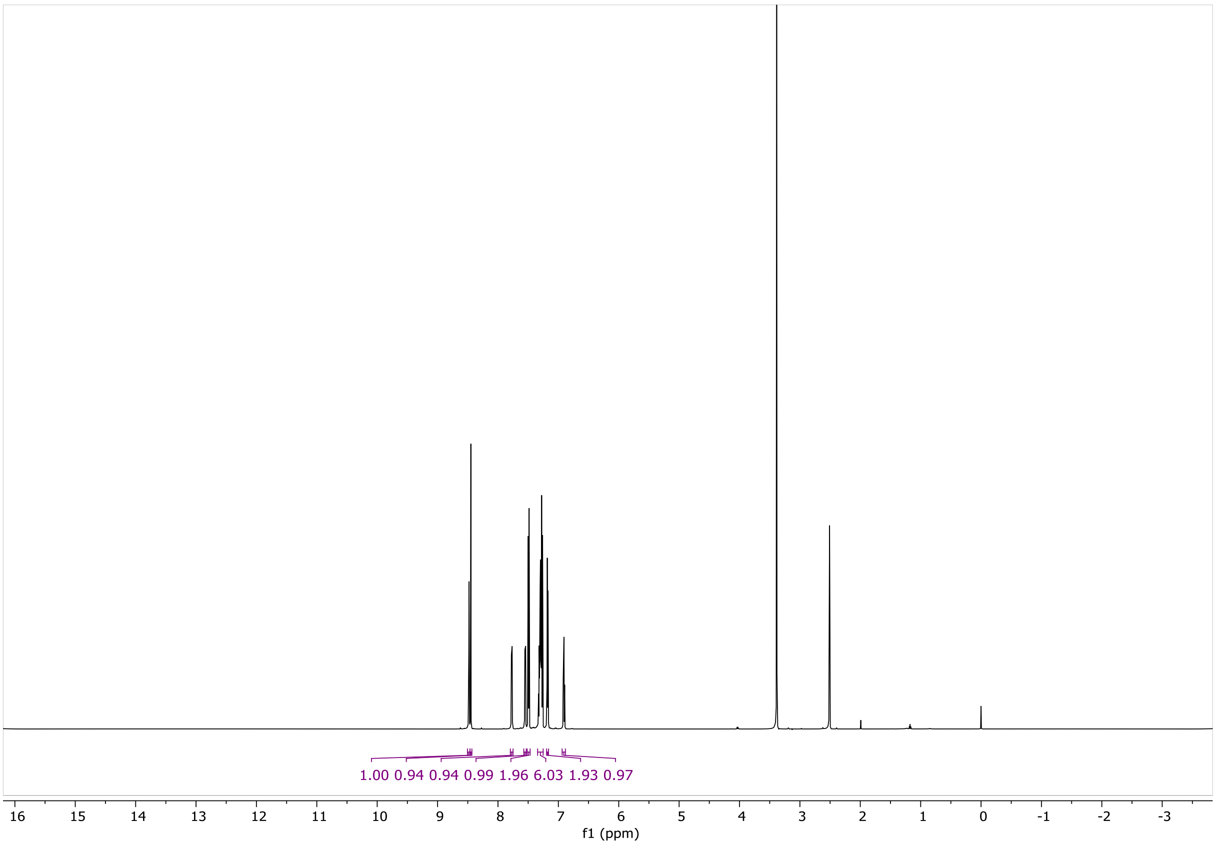

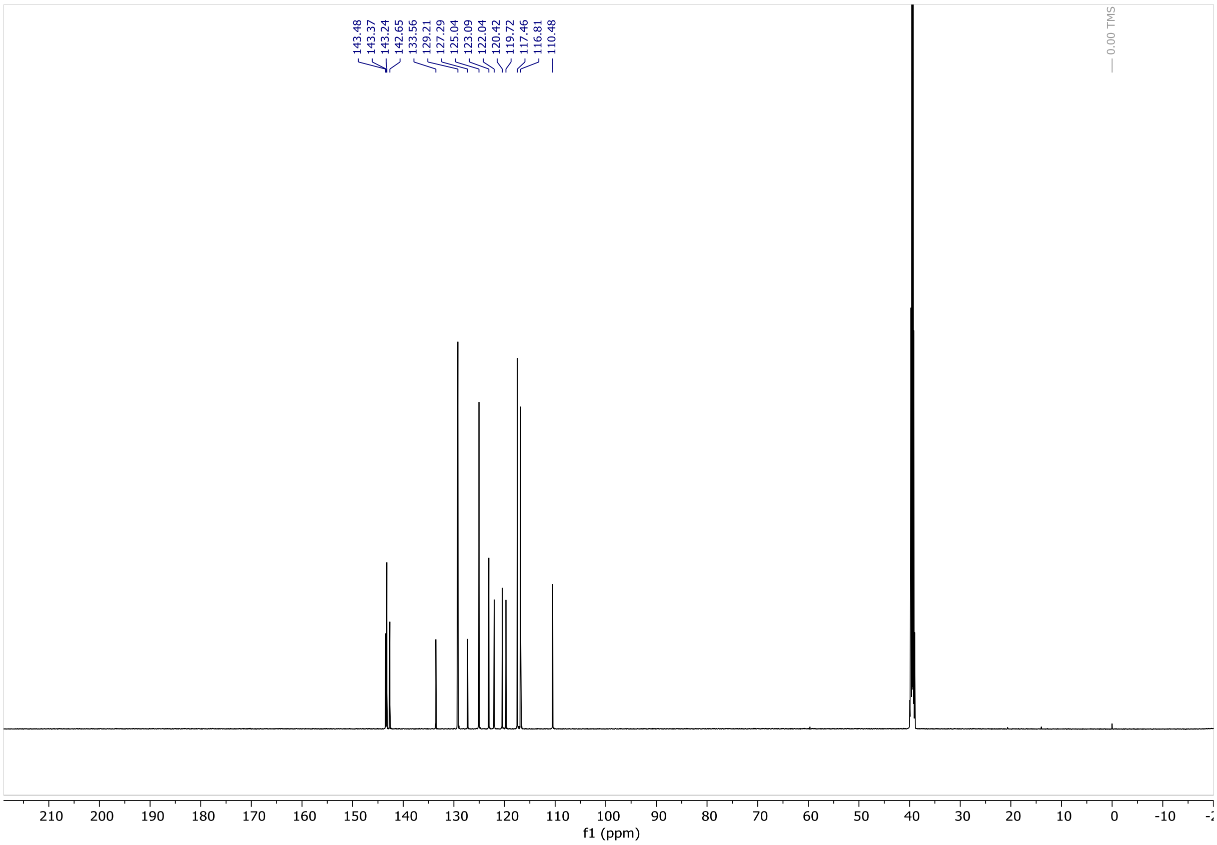

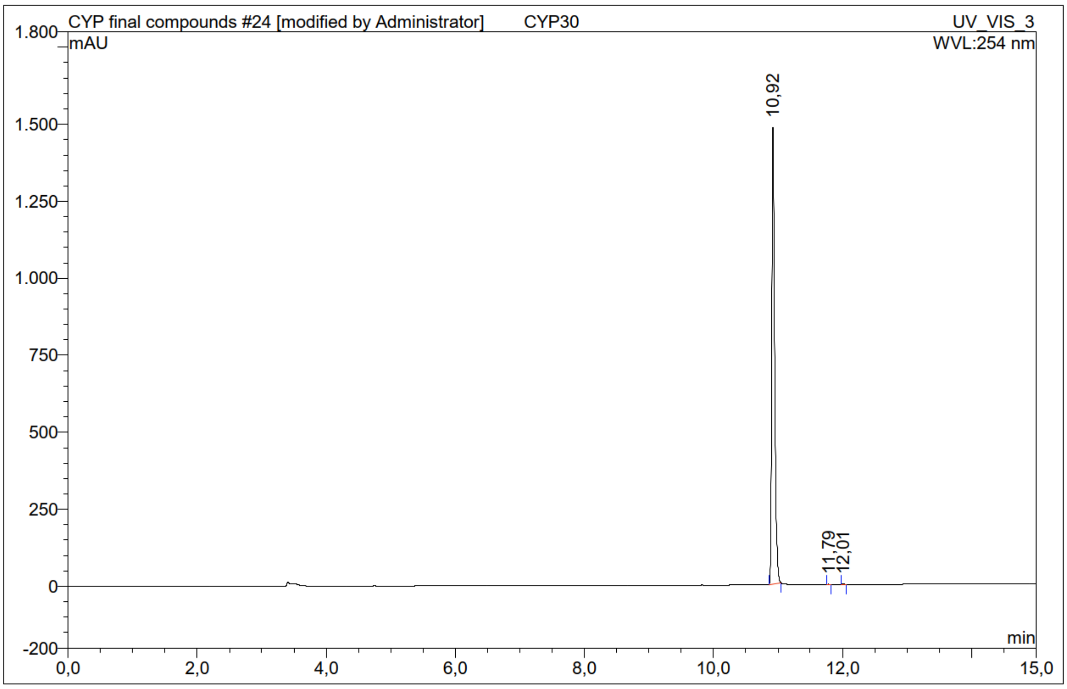

No.	Ret.Time min	Peak Name	Height mAU	Area mAU*min	Rel.Area %	Amount	Resolution(EP)
1	10,92	n.a.	1482,004	79,268	99,91	n.a.	11,73
2	11,79	n.a.	0,639	0,024	0,03	n.a.	3,24
3	12,01	n.a.	1,021	0,045	0,06	n.a.	n.a.
Total:			1483,665	79,337	100,00	0,000	

N-(4-(1H-indol-1-yl)phenyl)pyridin-4-amine (1e)

A flame-dried vial was charged with 4-aminopyridine (56 mg, 0.6 mmol), **2b** (136 mg, 0.5 mmol), tBuXPhos Pd G3 (20 mg, 0.025 mmol), tBuXPhos (11 mg, 0.025 mmol), and sodium t-butoxide (96 mg, 1 mmol). It was sealed and evacuated/backfilled with argon three times. 5 mL of anhydrous tBuOH was added and reaction was stirred at 70 °C overnight. Solvent was removed and the residue was partitioned between 5 mL of water and 10 mL of EtOAc. Aqueous was extracted 2 × 10 mL of EtOAc. Combined organics were dried with anhydrous Na_2SO_4 and striped of solvent. Remaining residue was purified by column chromatography on silica eluting MeOH / EtOAc (0:100 → 5:95) + 2% TEA to afford the title compound as a white solid (61 mg, 43%).

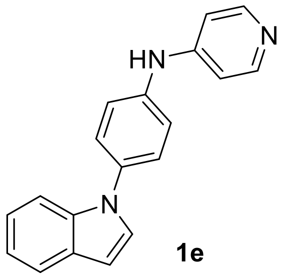

1e

^{1}H NMR (600 MHz, DMSO-*d*$_6$) δ 8.96 (s, 1H), 8.25 – 8.22 (m, 2H), 7.66 (dt, *J* = 7.8, 1.0 Hz, 1H), 7.61 (d, *J* = 3.2 Hz, 1H), 7.56 – 7.52 (m, 3H), 7.40 – 7.37 (m, 2H), 7.19 (ddd, *J* = 8.2, 7.0, 1.2 Hz, 1H), 7.12 (ddd, *J* = 7.9, 7.0, 1.0 Hz, 1H), 6.99 – 6.96 (m, 2H), 6.68 (dd, *J* = 3.2, 0.8 Hz, 1H). ^{13}C NMR (151 MHz, DMSO-*d*$_6$) δ 150.1, 149.8, 138.9, 135.2, 133.4, 128.7, 128.4, 124.9, 122.1, 120.8, 120.8, 120.0, 110.2, 109.3, 103.1. HRMS (MALDI): *m/z* calculated for $[M+H]^+$ 286.13387, found 286.13414.

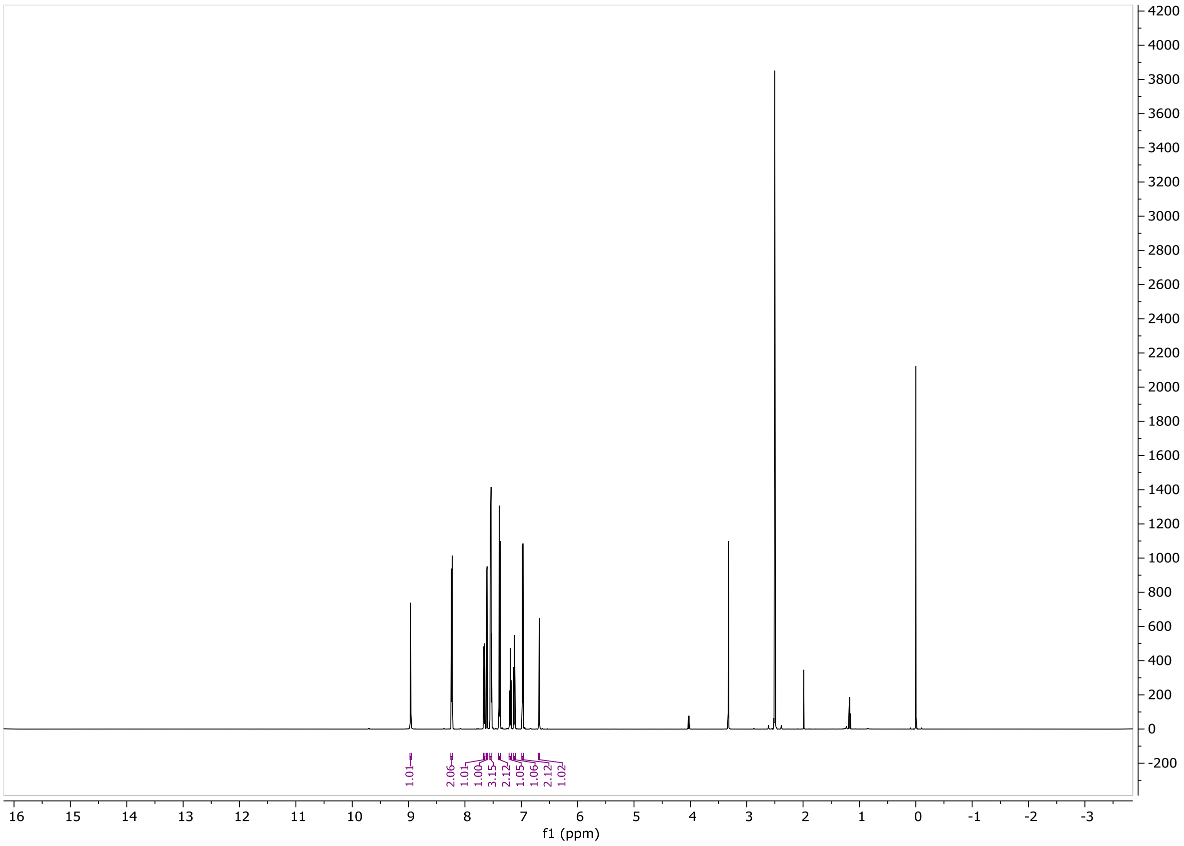

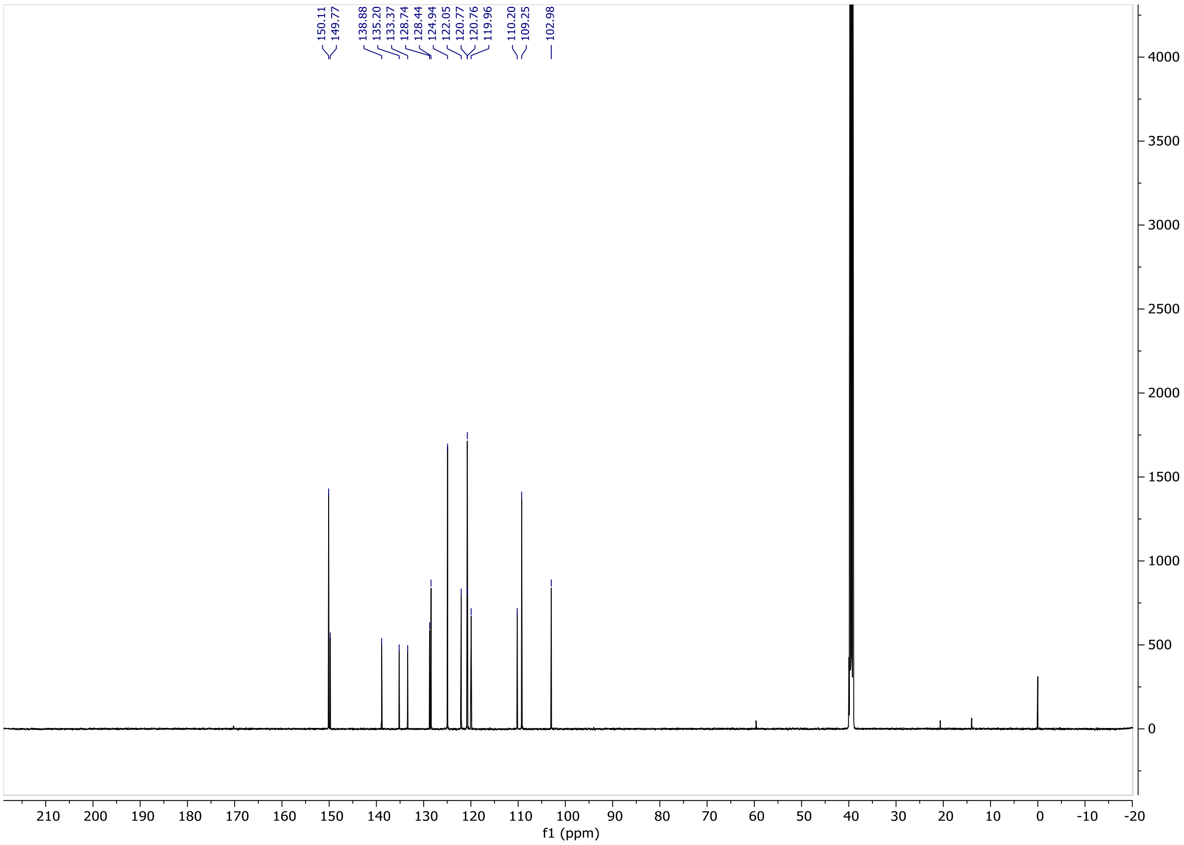

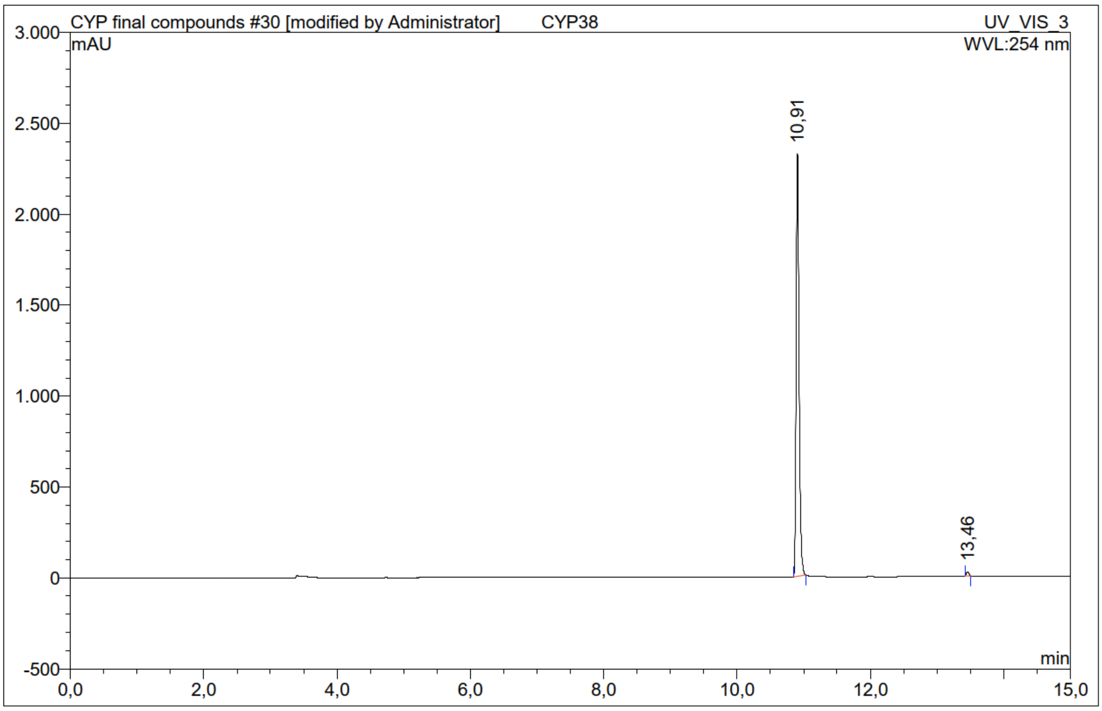

No.	Ret.Time min	Peak Name	Height mAU	Area mAU*min	Rel.Area %	Amount	Resolution(EP)
1	10,91	n.a.	2325,087	123,611	99,16	n.a.	32,32
2	13,46	n.a.	23,108	1,046	0,84	n.a.	n.a.
Total:			2348,194	124,657	100,00	0,000	

S2. Binding spectra and CYP IC50 curves

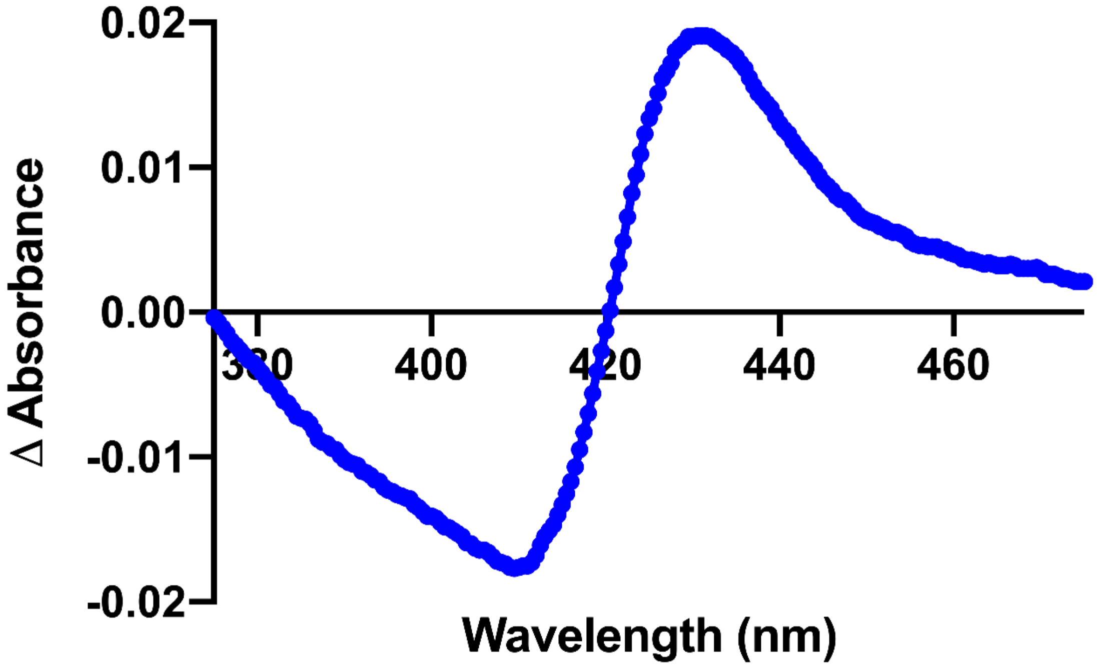

Difference spectrum of cytochrome P450 17A1 with vs. without compound **1a** (2 µM). All compounds are type II and display the same shift as the example above.

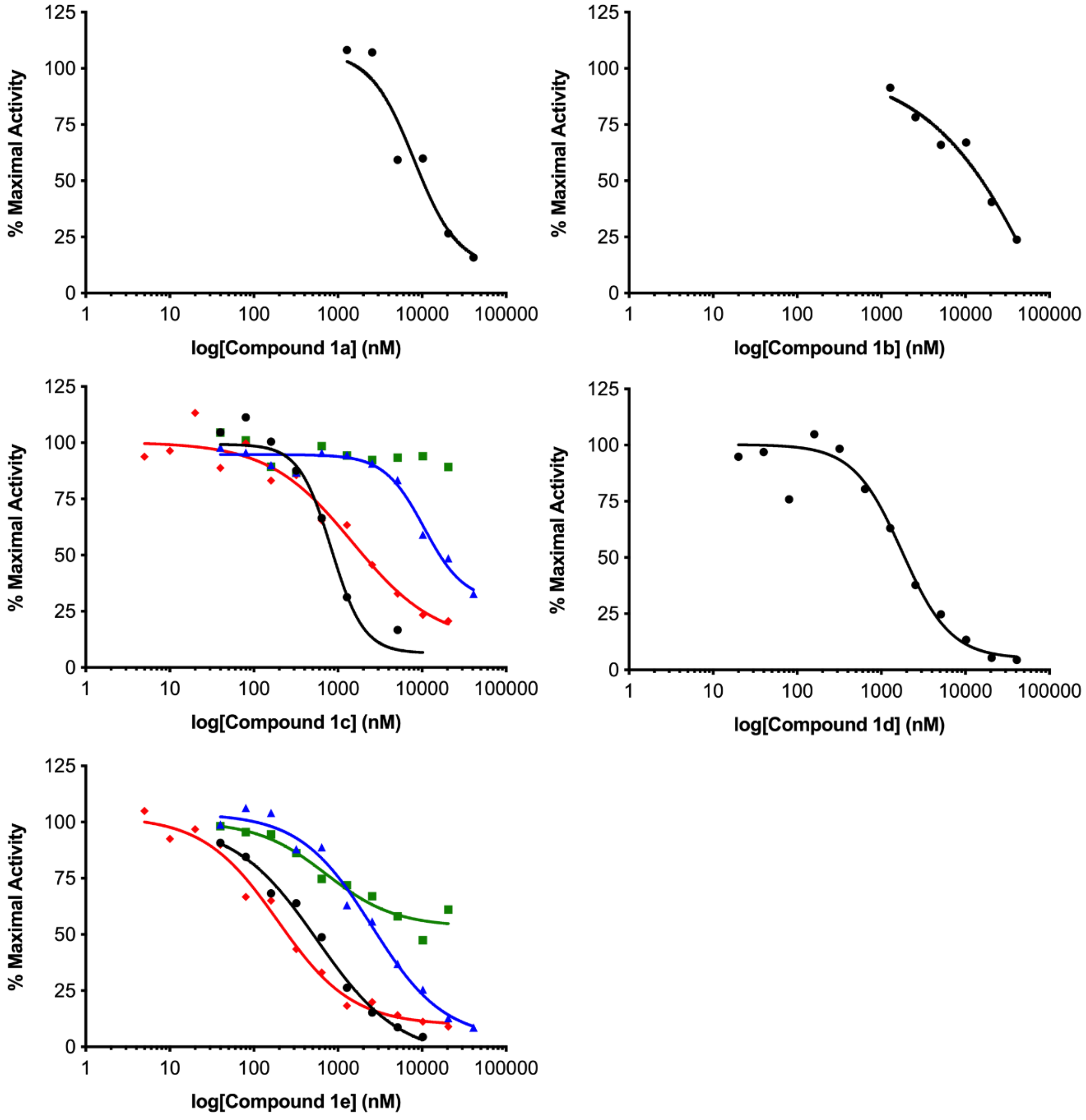

IC50 curves for compounds inhibiting CYP17A1 progesterone 17-hydroxylation (black circles), CYP21A2 progesterone 21-hydroxylation (red diamonds), CYP3A4 nifedipine metabolism (green squares), and CYP2D6 quinidine metabolism (blue triangles).

S3. Compound dose-response (GR$_{50}$) in PC-3 cells

ABT

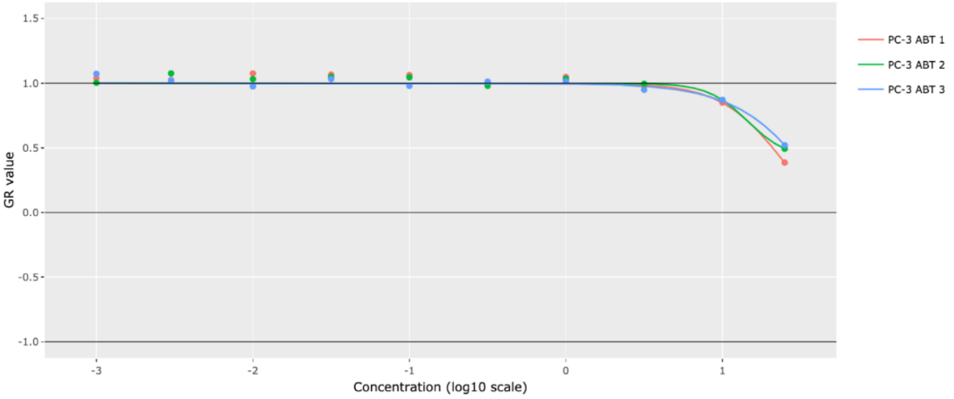

5-FU

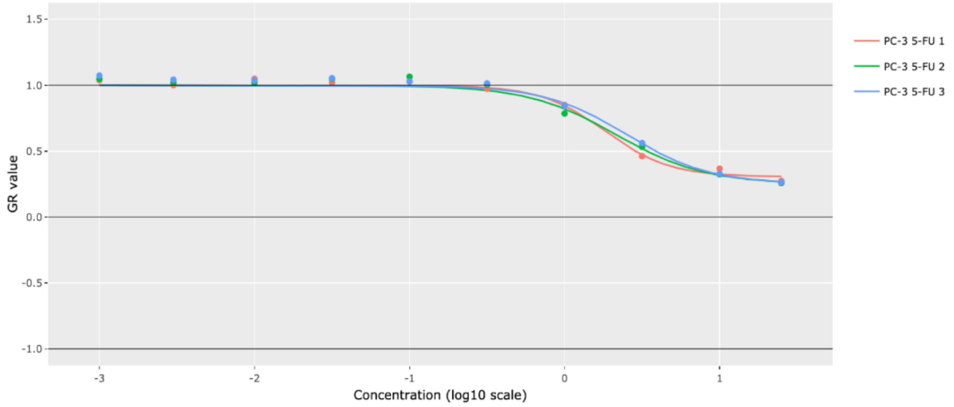

Compound 1a

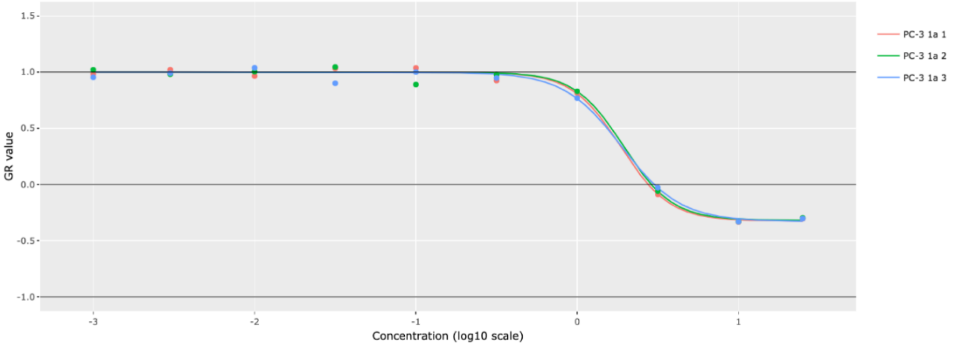

Compound 1b

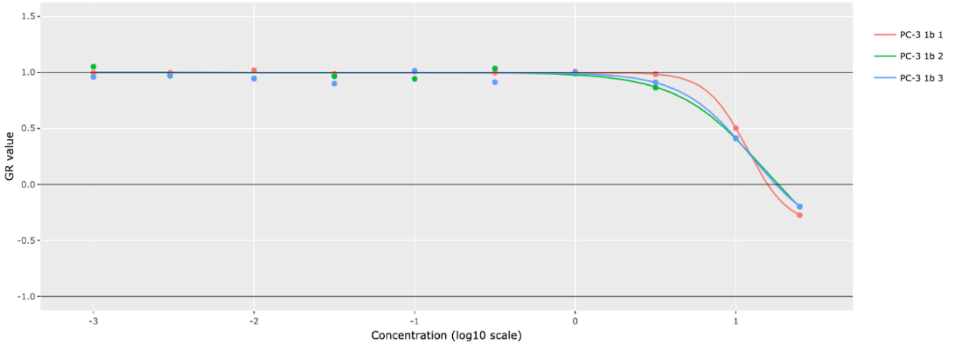

Compound 1c

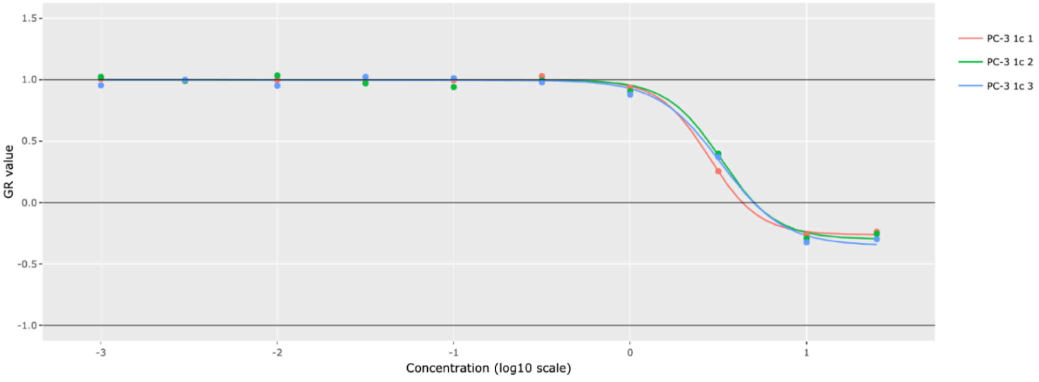

Compound 1d

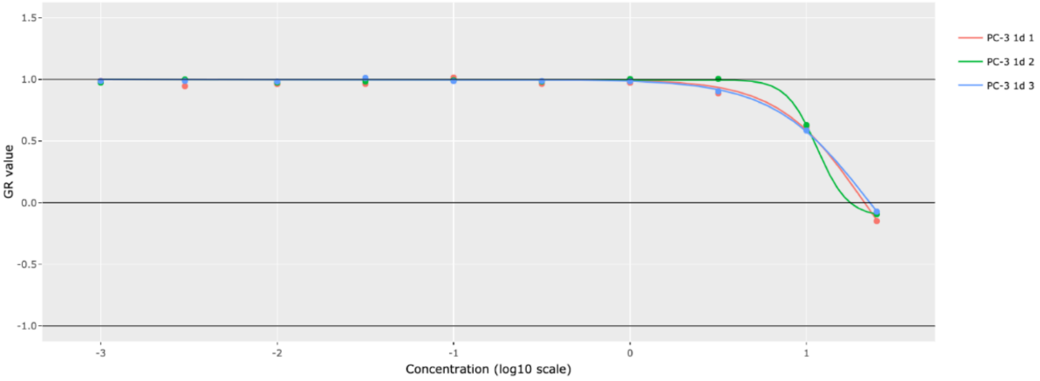

Compound 1e

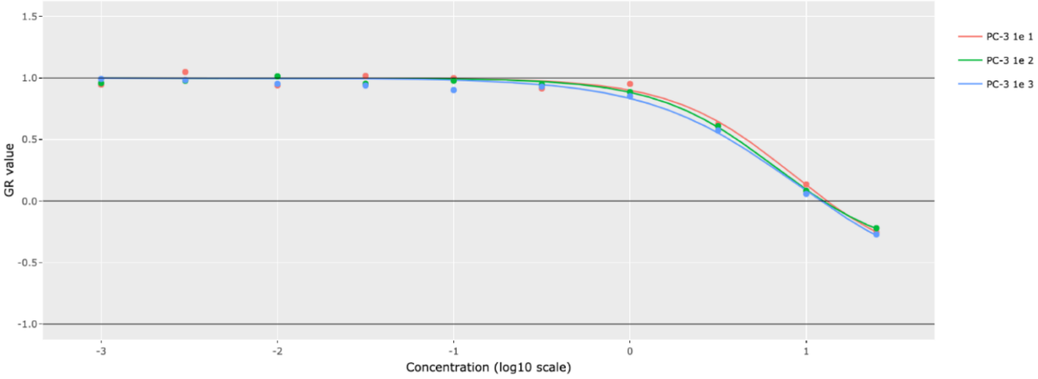

Cell Line	Treatment	GR50 (µM)
PC-3	ABT	23.7
PC-3	5-FU	3.4
PC-3	1a	1.6
PC-3	1b	9.2
PC-3	1c	2.6
PC-3	1d	11.9
PC-3	1e	4.1

Half maximal growth rate inhibition (GR50) values for all compounds in this study measured in the overall most responsive PC-3 cell line. Growth rate inhibition (GR) for dilution series (0-25 ◎M) of ABT, 5-FU, and compounds 1a–1e were calculated and plotted. Plots of three replicate dilution series, fitted dose–response curves, and calculated GR50 values (n=3) as given in the graphs and table were produced using the GRcalculator (Clark, N.A. et al. 2017)

S4. Docking poses

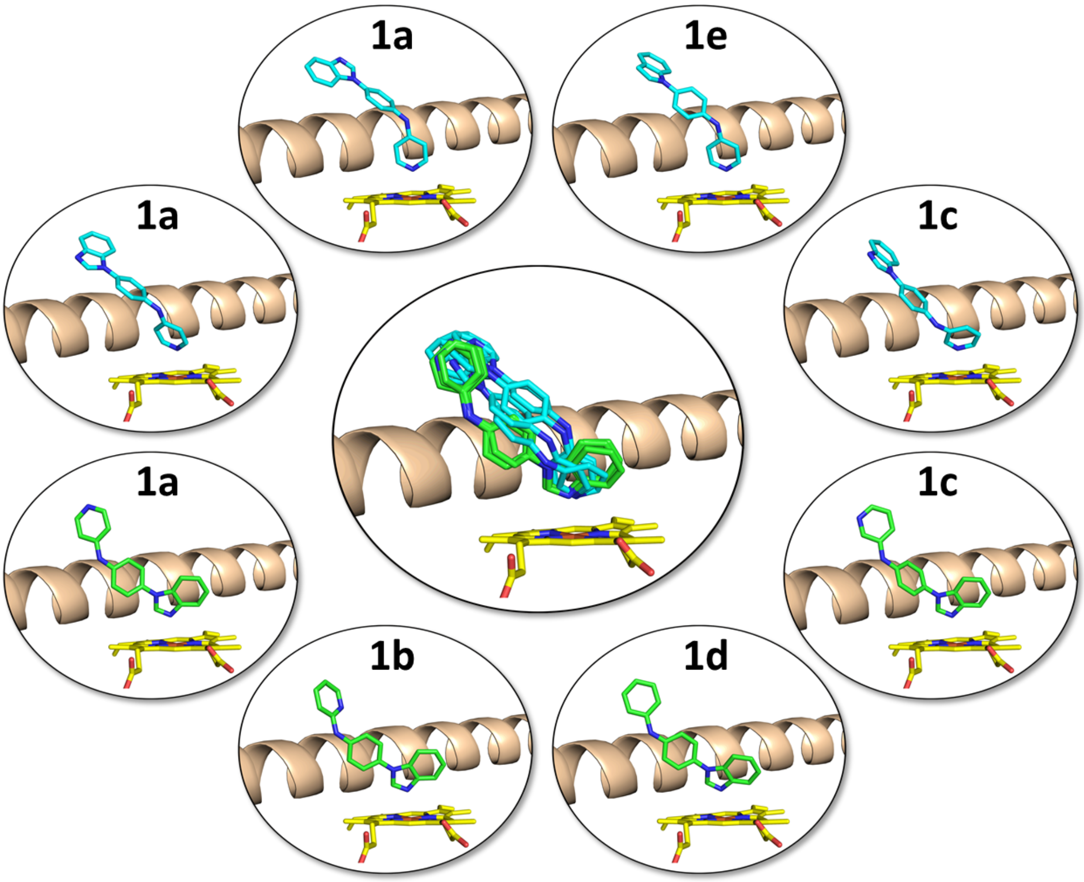

GOLD docking poses of compounds **1a**–**1e**. In poses with pyridine coordinating the iron in the heme group carbon atoms are colored cyan, in the remaining poses carbon atoms are colored green, nitrogen atoms are colored blue in all poses. For the protein, only the Helix I and the heme group are shown.

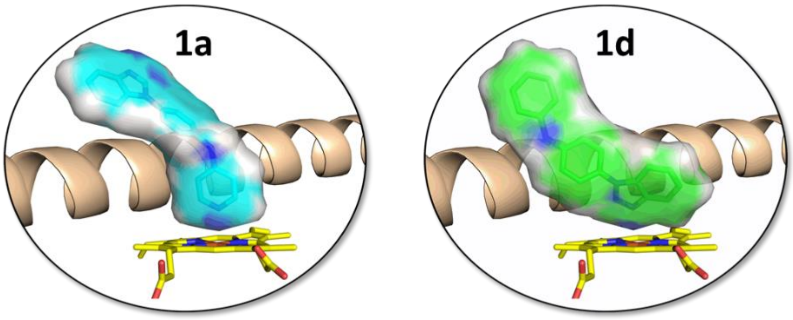

GOLD docking poses of compounds **1a** and **1d** illustrating the principal different binding modes of poses with the pyridine nitrogen coordinating the heme group (**1a**) and with the fused heterocyclic ring system coordinating the heme group (**1d**).
